# Solar Fuel Synthesis Using a Semiartificial Colloidal Z-Scheme

**DOI:** 10.1021/jacs.4c11827

**Published:** 2024-10-16

**Authors:** Yongpeng Liu, Ariffin Bin Mohamad Annuar, Santiago Rodríguez-Jiménez, Celine Wing See Yeung, Qian Wang, Ana M. Coito, Rita R. Manuel, Inês A. C. Pereira, Erwin Reisner

**Affiliations:** † Yusuf Hamied Department of Chemistry, University of Cambridge, Cambridge CB2 1EW, U.K.; ‡ Instituto de Tecnologia Química e Biológica António Xavier (ITQB NOVA), Universidade NOVA de Lisboa, Av. da República, 2780-157 Oeiras, Portugal

## Abstract

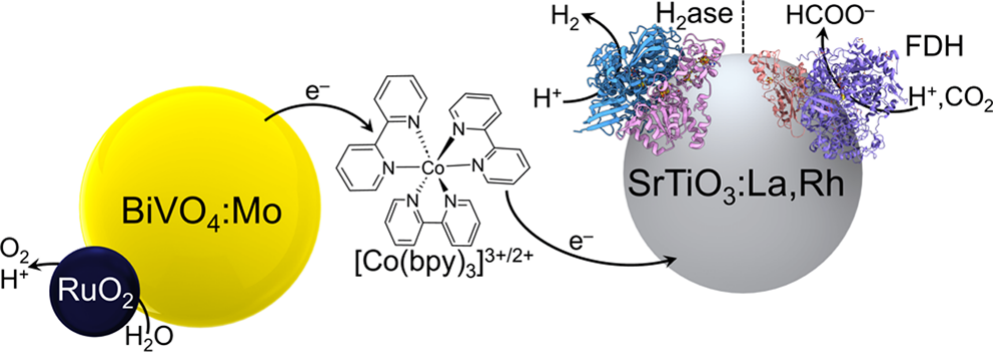

The integration of enzymes with semiconductor light absorbers in semiartificial photosynthetic assemblies offers an emerging strategy for solar fuel production. However, such colloidal biohybrid systems rely currently on sacrificial reagents, and semiconductor–enzyme powder systems that couple fuel production to water oxidation are therefore needed to mimic an overall photosynthetic reaction. Here, we present a Z-scheme colloidal enzyme system that produces fuel with electrons sourced from water. This “closed-cycle” semiartificial approach utilizes particulate SrTiO_3_:La,Rh and BiVO_4_:Mo (light absorbers), hydrogenase or formate dehydrogenase (cocatalyst), and a molecular cobalt complex (a redox mediator). Under simulated solar irradiation, this system continuously generates molecular hydrogen or formate, while co-producing molecular oxygen for 10 h using only sunlight, water, and carbon dioxide as inputs. In-depth analysis using quartz crystal microbalance, photoelectrochemical impedance spectroscopy, transient photocurrent spectroscopy, and intensity-modulated photovoltage spectroscopy provides mechanistic understanding and characterization of the semiconductor–enzyme hybrid interface. This study provides a rational platform to assemble functional semiartificial colloidal Z-scheme systems for solar fuel synthesis.

## Introduction

Storing solar energy as chemical fuels and feedstocks provides a means to advance sustainable technologies.^1^ Among emerging solar energy conversion approaches—photovoltaic-electrolysis, photoelectrochemistry, and photochemistry—the latter stands out due to its cost-effectiveness, device simplicity, and scalability.^2^ Solar-powered hydrogen (H_2_) production from water and carbon dioxide (CO_2_) reduction coupled to water oxidation to oxygen (O_2_) are particularly attractive solar fuel reactions (known as artificial photosynthesis).^3^ These processes usually employ semiconductors as light absorbers along with cocatalysts for specific chemical half-reactions. However, achieving an efficient overall reaction on a single light absorber faces challenges, notably in balancing solar light absorption (requiring a narrow band gap) and producing high-energy photogenerated charges (requiring a large band gap).^4^

To address this issue, a photosynthesis-inspired Z-scheme system utilizes two light absorbers—a semiconductor to drive the reductive chemistry and a semiconductor for the oxidation reaction.^5^ This artificial Z-scheme concept has been significantly advanced since its early development,^6^ but some key challenges such as finding efficient light absorbers, facilitating charge mediation between the two semiconductors, and developing selective cocatalysts persist, hindering progress for this technology.

Various semiconductors, including oxides,^7^ sulfides,^8^ and nitrides,^9^ have been explored as potential light absorbers in Z-scheme systems. SrTiO_3_ stands out as a model semiconductor for photochemical systems due to its high-energy conduction band edge, favorable for driving both proton and CO_2_ reduction.^10^ However, its wide band gap limits its light absorption to the ultraviolet (UV) region, and extensive efforts have thus focused on doping SrTiO_3_ with various elements to fine-tune its optoelectronic properties. For instance, Rh dopants can substitute Ti ions, creating intraband gap states that enable visible light absorption.^11^ Co-doping with La atoms for Sr sites further controls the valence states, leading to the state-of-the-art SrTiO_3_:La,Rh semiconductors for photoreductions.^9,12,13^ Among oxidative semiconductors, BiVO_4_ offers distinct advantages over TiO_2_ and WO_3_ for O_2_ evolution due to its intrinsic catalytic activity, narrower band gap suitable for visible light absorption, and low-energy conduction band edge that prevents competing reduction reactions in a Z-scheme configuration.^14,15^ Doping BiVO_4_ with elements such as Mo and W increases charge separation efficiency, thereby enhancing its photocatalytic activity.^16^

Establishing efficient electron transfer between the two semiconductors presents an enormous challenge for constructing a functional Z-scheme system. Natural photosynthesis employs a Z-scheme with the cytochrome *b*_6_f complex to shuttle electrons between photosystem I and photosystem II, enabling the overall oxidation of water to O_2_ and the fixation of CO_2_ to carbohydrates.^17^ In artificial Z-scheme systems, electron shuttles are categorized into soluble electron mediators (such as IO_3_^–^/I^–^, Fe^3+/2+^, and Co^3+/2+^)^7,14,18^ and solid-state mediators (including Au, graphene, and metal oxides).^8,13,19^ Among soluble electron mediators, IO_3_^–^/I^–^ is more effective in alkaline conditions with pH greater than 9, and Fe^3+/2+^ is only stable in acidic conditions with pH below 2.5. Molecular Co^3+/2+^ complexes exhibit functionality across a wide pH range, with optimal activity observed at neutral pH. Notably, molecular Co^3+/2+^ complexes have already demonstrated exceptional performance as redox couples for dye-sensitized solar cells, surpassing traditional I^–^/I^3–^ redox shuttles and achieving power conversion efficiencies exceeding 13%.^20,21^

In a Z-scheme system aimed at catalyzing H_2_ evolution and CO_2_ reduction reactions, loading cocatalysts onto reductive semiconductors is essential for enabling efficient and selective solar fuel synthesis. Various synthetic catalysts, including Pt,^8^ Ru,^14^ Au,^22^ and molecular 3d transition complexes,^12^ have been extensively studied. However, achieving selective CO_2_ conversion, especially toward products in the liquid phase, remains a significant challenge in this field.^5^ In contrast to synthetic catalysts, nature has evolved dedicated enzymes to catalyze specific physiological processes. For instance, hydrogenases (H_2_ases) and formate dehydrogenases (FDHs) facilitate the respective interconversion between proton and H_2_, CO_2_ and formate at the thermodynamic potential under mild conditions.^23,24^ The integration of enzymes and light absorbers in a semiartificial approach has emerged as a promising research direction to leverage the performance strength of enzymes. However, a key challenge that persists is the reliance on sacrificial reagents for the functionality of colloidal semiartificial systems.^25,26^ By mediating electron transfer between two semiconductors, a Z-scheme configuration can effectively preserve high-energy electrons and holes for specific reactions. This capability holds significant potential for achieving overall reactions without the need for sacrificial reagents in semiartificial photosynthesis. The application of enzymes as cocatalysts for selective solar fuel synthesis has been reported in photoelectrochemical tandem cells,^27,28^ but interfacing enzymes with colloidal Z-scheme systems to couple water oxidation to fuel production remains a challenge.

In this study, we present a semiartificial colloidal photosynthetic Z-scheme system for solar fuel synthesis by coupling water oxidation to H_2_ production or CO_2_ reduction. The assembly of H_2_ase or FDH enzymes with SrTiO_3_:La,Rh|[Co(bpy)_3_]^3+/2+^|BiVO_4_:Mo|RuO_2_ (bpy = 2,2′-bipyridine) enables overall water splitting or CO_2_ reduction to formate coupled to O_2_ evolution, respectively. A benefit of using a semiconductor suspension system is their operation in bulk (3D) solution without the complex device construction process and confined two-dimensional (2D) surface in photoelectrodes,^27,28^ artificial leaves,^29^ and photocatalyst sheets.^12,13^ The semiconductor light absorbers were characterized by Mott–Schottky analysis to confirm the suitable band edge positions of SrTiO_3_:La,Rh and BiVO_4_:Mo. The adsorption of enzymes onto the reductive semiconductor was studied by using a quartz crystal microbalance (QCM). Furthermore, we investigated the charge carrier dynamics of the semiartificial Z-scheme systems using photoelectrochemical techniques such as photoelectrochemical impedance spectroscopy (PEIS), intensity-modulated photovoltage spectroscopy (IMVS), and transient photocurrent spectroscopy (TPC).

## Results and Discussion

### Selection and Characterizations

[NiFeSe]-H_2_ase and [W]-FDH from *Desulfovibrio vulgaris* Hildenborough (*Dv*H) were chosen as model enzymes due to their selective and reversible catalysis (very low overpotential requirement) for H_2_ evolution and CO_2_-to-formate conversion under mild conditions, respectively. Furthermore, the enzymes attach strongly to metal oxides in an electroactive configuration, and display a moderate tolerance to O_2_.^23,24,30^ For the fuel-forming (reduction) semiconductor, SrTiO_3_:La,Rh was selected due to its high conduction band edge, excellent dispersity in solution, and visible light absorption properties.^11,31^ RuO_2_ was loaded as the cocatalyst onto the oxidation semiconductor BiVO_4_:Mo, specifically designed for water oxidation.^12,13^

**Figure 1:**
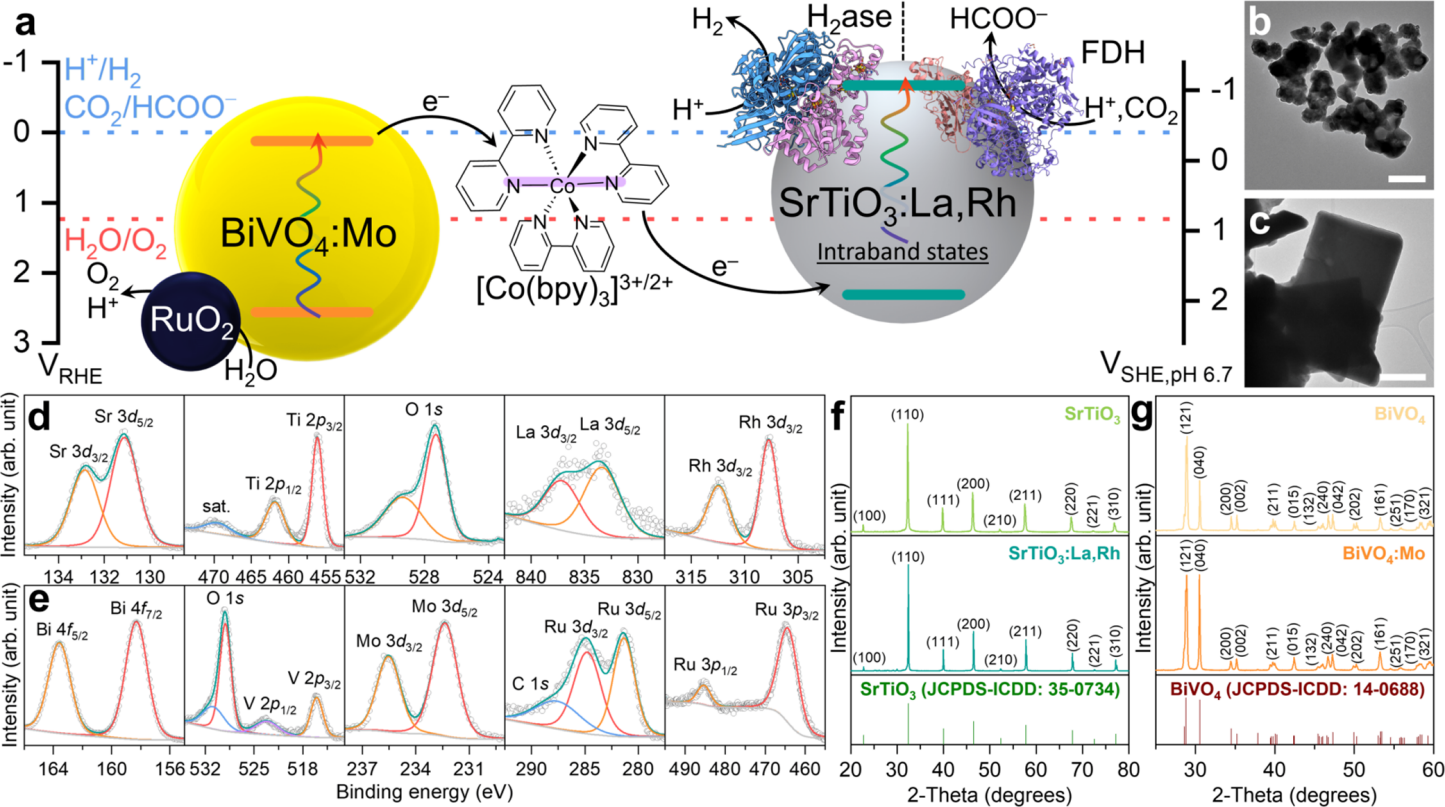
(a) Schematic illustration of a semiartificial colloidal Z-scheme system with H_2_ase (PDB: 5jsh) and FDH (PDB: 6sdv). Estimated band positions for SrTiO_3_:La,Rh and BiVO_4_:Mo are given with redox potentials for [Co(bpy)_3_]^3+/2+^, H^+^/H_2_, CO_2_/HCOO^–^, and H_2_O/O_2_. TEM images of (b) SrTiO_3_:La,Rh and (c) BiVO_4_:Mo|RuO_2_, scale bar: 500 nm. Curve-fitted XPS spectra of (d) SrTiO_3_:La,Rh and (e) BiVO_4_:Mo|RuO_2_. Powder XRD patterns for (f) SrTiO_3_ and SrTiO_3_:La,Rh with the corresponding diffraction patterns for SrTiO_3_ (JCPDS-ICDD: 35–0734) and (g) BiVO_4_ and BiVO_4_:Mo with the corresponding diffraction patterns for BiVO_4_ (JCPDS-ICDD: 14–0688).

Figure 1a depicts the proposed semiartificial colloidal photosynthetic Z-scheme for solar H_2_ production or solar CO_2_-to-formate conversion using water as the electron donor. Tauc plots (Figure S1) and Mott–Schottky analysis (Figure S2) confirmed the band diagram of SrTiO_3_:La,Rh and BiVO_4_:Mo, where both the conduction band edge (*E*_C_) and the valence band edge (*E*_V_) of SrTiO_3_:La,Rh are higher than those of BiVO_4_:Mo, and the band gaps of the two semiconductors overlap,^9,12,13^ illustrating a suitable energy band alignment between the two semiconductors for constructing a Z-scheme system. Cyclic voltammetry analysis of the [Co(bpy)_3_]^3+/2+^ complex revealed a half-wave potential (*E*_1/2_) of 0.70 V *vs* the reversible hydrogen electrode (RHE).^14^ This value falls within the band gaps of both SrTiO_3_:La,Rh and BiVO_4_:Mo, showing the suitability of [Co(bpy)_3_]^3+/2+^ as an electron mediator for the proposed Z-scheme system, and is in agreement with the literature.^9,12,14^

Upon irradiation, photogenerated holes in the valence band of BiVO_4_:Mo transfer to RuO_2_ for the oxygen evolution reaction (OER), while photogenerated electrons in the conduction band reduce [Co(bpy)_3_]^3+^ to [Co(bpy)_3_]^2+^. Meanwhile, photogenerated holes in the intraband states of SrTiO_3_:La,Rh oxidize [Co(bpy)_3_]^2+^ back to [Co(bpy)_3_]^3+^, while the photogenerated electrons in the conduction band either reduce protons to H_2_ (when paired with H_2_ase) or reduce CO_2_ to formate (when paired with FDH). The regeneration of the [Co(bpy)_3_]^3+/2+^ redox couple allows the spatial separation of photogenerated electrons and holes in the two semiconductors, thereby enhancing charge separation efficiency and preserving high-energy photogenerated charges for catalyzing the corresponding reactions.

Scanning electron microscopy (SEM) and transmission electron microscopy (TEM) revealed the morphology of SrTiO_3_:La, Rh nanoparticles and RuO_2_ loaded BiVO_4_:Mo nanoplates (Figures 1b–c and S3–S8). The presence of individual elements of SrTiO_3_:La,Rh and BiVO_4_:Mo was confirmed through energy-dispersive X-ray (EDX) mapping (Figures S9–S11) and X-ray photoelectron spectroscopy (XPS, Figure 1d–e). To identify lattice changes induced by doping, powder X-ray diffraction (XRD) analysis was conducted on both pristine and doped metal oxide semiconductors. The XRD pattern of SrTiO_3_:La,Rh, compared to pristine SrTiO_3_, retains the cubic structure (Figure 1f) but exhibits a peak shift toward higher angles (Figure S12a). This shift indicates a successful doping process, reflecting changes in lattice parameters due to the substitution of Sr^2+^ (0.144 nm) and Ti^4+^ (0.060 nm) ions with La^3+^ (0.136 nm) and Rh^3+^ (0.068 nm) ions.^32^ Additionally, the XRD pattern of BiVO_4_:Mo corresponds to the monoclinic phase of BiVO_4_, with no evidence of secondary phases such as MoO*_x_* (Figure 1g). The observed peak shift confirms the successful substitution of Mo^6+^ ions (0.059 nm) with V^5+^ ions (0.054 nm) within the BiVO_4_ crystal lattice (Figure S12b).^33^ These peak shifts are expected, as doping alters the *d*-spacing due to differences in the ionic radii of the dopant and host atoms. The absorption profiles of SrTiO_3_:La,Rh and BiVO_4_:Mo were characterized by ultraviolet–visible (UV–vis) spectroscopy (Figure S13).

With the aim of maintaining the *in vitro* activity of H_2_ases and FDHs under benign conditions, the [Co(bpy)_3_]^3+/2+^ redox mediator was identified as an ideal candidate due to its outstanding activity at neutral pH. Water-soluble [Co(bpy)_3_]SO_4_ was synthesized following a reported procedure^14,34^ (reaction scheme and photograph in Figures S14 and S15) and characterized using elemental analysis, mass spectrometry, proton nuclear magnetic resonance (^1^H NMR) spectroscopy (Experimental Section, Figure S16), attenuated total reflectance Fourier-transform infrared (ATR-FTIR) spectroscopy (Figure S17), and UV–vis spectroscopy (Figure S18).

### Photocatalytic Half Reactions

We started the stepwise construction of a functional Z-scheme system by conducting Z-scheme half reactions separately on the reduction and oxidation semiconductors, respectively, with the counterreaction balanced by the stoichiometric conversion of the [Co(bpy)_3_]^3+/2+^ redox mediator. In a colloidal system, photocatalytic H_2_ production was established by interfacing H_2_ase (20 pmol) with SrTiO_3_:La,Rh (1 mg) in a CO_2_-saturated aqueous solution (1 mL) containing NaHCO_3_ (0.1 M) and [Co(bpy)_3_]SO_4_ (0.5 μmol) under simulated AM 1.5G irradiation at 25 °C. The solutions with H_2_ase contained CO_2_-saturated NaHCO_3_ to match the anaerobic conditions using FDH (see below).

**Figure 2:**
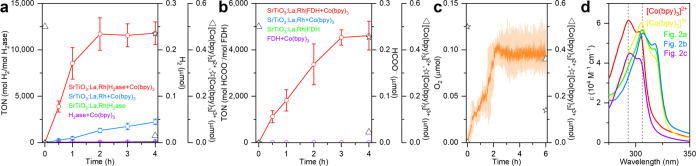
Z-scheme half reactions with SrTiO_3_:La,Rh coupled to stoichiometric [Co(bpy)_3_]^2+^ oxidation using (a) H_2_ase for photocatalytic proton reduction to H_2_ and (b) FDH for photocatalytic reduction of CO_2_ to formate. (c) Z-scheme half reaction with BiVO_4_:Mo|RuO_2_ coupled to stoichiometric [Co(bpy)_3_]^3+^ reduction for photocatalytic water oxidation to O_2_. (d) UV absorption spectra of aqueous standard [Co(bpy)_3_]^3+/2+^ solutions and post photocatalysis solutions. Conditions: a CO_2_-saturated aqueous solution (1 mL, pH 6.7) containing NaHCO_3_ (0.1 M), [Co(bpy)_3_]^3+/2+^ (0.5 μmol), SrTiO_3_:La,Rh (1 mg), BiVO_4_:Mo|RuO_2_ (1 mg), H_2_ase (20 pmol), FDH (50 pmol), AM 1.5G irradiation, 600 rpm stirring, 25 °C. Error bars represent the standard deviation for a sample size of 3.

The time dependent H_2_ evolution (Figure 2a and Table S1) displayed a linear increase in the first hour, saturating after 2 h at 0.234 ± 0.035 μmol H_2_, corresponding to a turnover number (TON) of 11,700. The TON is determined by the ratio of moles product (H_2_) to moles enzyme (H_2_ase).^35^ The *in vitro* activity of H_2_ase ceased after 2 h because of the stoichiometric consumption of [Co(bpy)_3_]SO_4_. Considering that H_2_ evolution is a two-electron transfer process, the saturated H_2_ yield corresponds to 0.468 μmol of consumed electrons and closely matches the 0.5 μmol of [Co(bpy)_3_]^2+^ in the solution. This confirms that SrTiO_3_:La,Rh|H_2_ase assemblies can simultaneously reduce protons to H_2_ and oxidize [Co(bpy)_3_]^2+^ to [Co(bpy)_3_]^3+^, establishing the foundation for the reduction half reaction of a Z-scheme system. The depletion of [Co(bpy)_3_]^2+^ in the solution was confirmed by UV–vis spectroscopy (Figure 2d) using the quantification method based on the Beer–Lambert law: *A* = ε*cl*, where *A* is the absorbance, ε is the molar absorption coefficient (M^–1^ cm^–1^), *c* is the molar concentration (*M*), and *l* is the optical path length (cm) (see Experimental Section).^14,36^ This method is based on the principle that changes in the Co(bpy)_3_ valence state affect the π–π* transitions of ligands, leading to alterations in UV absorption. An increase in the central Co ion valence from 2+ to 3+ results in a bathochromic shift (red shift) in the absorption spectrum from 293 to 306 nm.^36−38^ The ε of [Co(bpy)_3_]SO_4_ was found to be 6.209 × 10^4^ M^–1^ cm^–1^, similar to the previous reports for [Co(bpy)_3_]SO_4_ (6.958 × 10^4^ M^–1^ cm^–1^)^14^ and [Co(bpy)_3_](ClO_4_)_2_ (4.2 × 10^4^ M^–1^ cm^–1^).^36^ After 4 h of photocatalysis, 0.47 μmol of [Co(bpy)_3_]^3+^ was produced, corresponding to a 94% conversion yield for Co^2+^ to Co^3+^. Kudo and co-workers previously identified Rh ions in SrTiO_3_:Rh as the active sites for [Co(bpy)_3_]^2+^ oxidation.^14^

Exclusion control experiments (Figure 2a and Table S1) were conducted by systematically removing individual components from the photocatalytic system. As depicted in Figure 2a, no photocatalytic H_2_ generation was observed in the absence of SrTiO_3_:La,Rh_4_. The absence of [Co(bpy)_3_] resulted in no H_2_ production, indicating that [Co(bpy)_3_]^2+^ is the exclusive electron donor in the system, and SrTiO_3_:La,Rh alone cannot catalyze overall water splitting. In the absence of H_2_ase, SrTiO_3_:La,Rh produced a minor amount of H_2_, reaching a yield of 0.04 ± 0.006 μmol of H_2_ over 4 h. This observation suggests that dopants in SrTiO_3_:La,Rh such as Rh sites can catalyze some H_2_ production.^39^

The reduction half reaction on SrTiO_3_:La,Rh|FDH assemblies in a CO_2_-saturated aqueous solution (1 mL) containing NaHCO_3_ (0.1 M), [Co(bpy)_3_]SO_4_ (0.5 μmol), SrTiO_3_:La,Rh (1 mg), and FDH (50 pmol) under simulated AM 1.5G irradiation resulted in a linear solar formate production that saturated at 4 h with 0.230 ± 0.031 μmol, corresponding to a TON_FDH_ of 4,614 (Figure 2b and Table S2). The two-electron transfer CO_2_-to-formate reaction consumed 0.454 μmol of photogenerated electrons, consistent with the initial 0.5 μmol of [Co(bpy)_3_]^2+^ in the solution and the 0.455 μmol of [Co(bpy)_3_]^3+^ measured after 4 h of photocatalysis (Figure 2d, Co^2+^ to Co^3+^ conversion yield: 91%). Notably, this solar formate production half reaction could not be achieved in the absence of any components (Figure 2b and Table S2), indicating that FDH is the sole catalyst in the system capable of reducing CO_2_. This study also demonstrates direct electron transfer between a SrTiO_3_-based semiconductor and enzymes, providing further support for the notion that metal oxides serve as excellent scaffolds for accommodating enzymes in their active orientations.^27−30^

The oxidation half reactions were conducted on BiVO_4_:Mo|RuO_2_ (1 mg) with [Co(bpy)_3_]_2_(SO_4_)_3_ (0.5 μmol) in a CO_2_-saturated aqueous solution (1 mL) containing NaHCO_3_ (0.1 M) under simulated AM 1.5G irradiation at 25 °C. As shown in Figure 2c, a linear increase in O_2_ production over 2.2 h is followed by saturation at around 0.1 μmol. Considering the four-electron water oxidation, approximately 0.4 μmol of photogenerated holes were consumed, indicating that the termination of O_2_ production was due to the depletion of [Co(bpy)_3_]^3+^. The conversion yield of Co^3+^ to Co^2+^ was found to be 72% (Figure 2d). By completing all three half reactions, we have confirmed the suitability of the SrTiO_3_:La,Rh|enzyme biohybrids for the reduction half reaction and BiVO_4_:Mo|RuO_2_ for the oxidation half reaction, thereby establishing the foundation for constructing a functional semiartificial Z-scheme system using [Co(bpy)_3_]^3+/2+^ as the redox mediator.

### Photosynthetic Z-Scheme Systems

**Figure 3:**
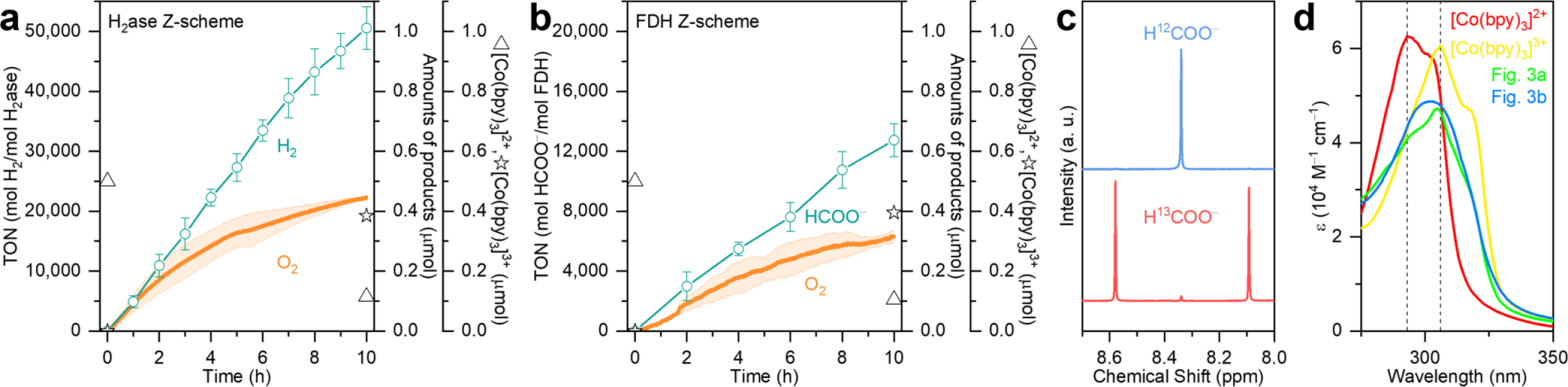
(a) Photocatalytic overall water splitting using the Z-scheme H_2_ase|SrTiO_3_:La,Rh|[Co(bpy)_3_]^3+/2+^|BiVO_4_:Mo|RuO_2_. (b) Photocatalytic reduction of CO_2_ to formate coupled with water oxidation using the Z-scheme FDH|SrTiO_3_:La,Rh|[Co(bpy)_3_]^3+/2+^|BiVO_4_:Mo|RuO_2_. (c) ^1^H NMR spectra of the photocatalysis solution containing ^12^CO_2_/NaH^12^CO_3_ (blue line) and ^13^CO_2_/NaH^13^CO_3_ (red line) after 10 h of irradiation on FDH|SrTiO_3_:La,Rh|[Co(bpy)_3_]^3+/2+^|BiVO_4_:Mo|RuO_2_. (d) UV absorption spectra of aqueous standard [Co(bpy)_3_]^3+/2+^ solutions and post photocatalysis solutions. Conditions: a CO_2_-saturated aqueous solution (1 mL, pH 6.7) containing NaHCO_3_ (0.1 M), [Co(bpy)_3_]SO_4_ (0.5 μmol), SrTiO_3_:La,Rh (1 mg), BiVO_4_:Mo|RuO_2_ (1 mg), H_2_ase (20 pmol), FDH (50 pmol), AM 1.5G irradiation, 600 rpm stirring, 25 °C. Error bars represent the standard deviation for a sample size of 3.

A semiartificial Z-scheme system for overall water splitting was subsequently developed using a colloidal suspension consisting of SrTiO_3_:La,Rh|H_2_ase powder and BiVO_4_:Mo|RuO_2_ powder dispersed in an aqueous solution containing [Co(bpy)_3_]SO_4_ (0.5 μmol), giving H_2_ase|SrTiO_3_:La,Rh|[Co(bpy)_3_]^3+/2+^|BiVO_4_:Mo|RuO_2_, in a CO_2_-saturated NaHCO_3_ (0.1 M) solution (1 mL, pH 6.7) under simulated AM 1.5G irradiation at 25 °C (Figure 3a and Table S3). Unlike the early termination observed in individual half reactions (Figure 2), the full Z-scheme system demonstrated the ability to generate solar H_2_ continuously over a period of 10 h in a nearly linear manner, accompanied by simultaneous solar O_2_ production.

This outcome supports our hypothesis (Figure 1a) that the [Co(bpy)_3_]^3+/2+^ redox couple, acting as the electron shuttle, is cycled by the photogenerated holes in SrTiO_3_:La,Rh and by the photogenerated electrons in BiVO_4_:Mo. The colloidal photosynthetic Z-scheme system achieved a H_2_ production yield of 1.01 ± 0.07 μmol (corresponds to an activity, based on the mass of the photocatalyst, of 50.6 ± 3.6 μmol g^–1^ h^–1^) and O_2_ production of 0.45 ± 0.004 μmol (corresponds to an activity of 22.4 ± 0.2 μmol g^–1^ h^–1^) in 10 h. The turnover frequency (TOF) for H_2_ reached 5,055 h^–1^ in 10 h (Table S4). The apparent quantum yield (AQY) at 420 nm and the solar-to-H_2_ energy conversion efficiency (STH) were found to be 0.8% and 0.007% (10 h), respectively. The molar ratio between H_2_ and O_2_ was nearly stoichiometric at approximately 2:0.89 at 10 h.^5,14^ In overall water splitting, separate gas evolution is particularly crucial for large-scale H_2_ production due to the formation of explosive H_2_ and O_2_ gas mixtures as well as requiring O_2_-free H_2_ for downstream use of H_2_ (*e.g.*, hydrogenation chemistry, use in fuel cells).^40^ However, this aspect is beyond the scope of our present research, which primarily focuses on demonstrating fundamental research in constructing colloidal biohybrids for photocatalysis and producing H_2_ on a μmol scale.

After 10 h of photocatalysis, the ratio of [Co(bpy)_3_]^2+^ to [Co(bpy)_3_]^3+^ was approximately 1 to 3.3 (Figure 3a,d), indicating the predominance of trivalent [Co(bpy)_3_] post photocatalysis. The turnover frequency for cycling [Co(bpy)_3_]^3+/2+^ in the overall water splitting is 0.52 h^–1^. Similar observations were reported by Kudo and colleagues, who noted a 1 to 9 ratio for [Co(phen)_3_]^2+^ to [Co(phen)_3_]^3+^ after long-term photocatalysis using [Co(phen)_3_]^3+/2+^ as the redox mediator for Z-scheme reactions.^14^ The authors also reported that the TOF of [Co(bpy)_3_]^3+/2+^ electron mediator with SrTiO_3_:Rh|Ru is 0.32 h^–1^ (without Sr excess) and 3.3 h^–1^ (with Sr excess).^14^

Semiartificial solar CO_2_-to-formate production coupled with O_2_ evolution was performed using the FDH|SrTiO_3_:La,Rh|[Co(bpy)_3_]^3+/2+^|BiVO_4_:Mo|RuO_2_ suspension in a CO_2_-saturated aqueous solution (1 mL) containing NaHCO_3_ (0.1 M) and [Co(bpy)_3_]SO_4_ (0.5 μmol) under simulated AM 1.5G irradiation at 25 °C (Figure 3b and Table S3). The overall reaction was sustained for 10 h, resulting in the production of 0.64 ± 0.05 μmol (31.9 ± 2.7 μmol g^–1^ h^–1^) of formate and 0.32 ± 0.02 μmol (15.8 ± 1.0 μmol g^–1^ h^–1^) of O_2_. The TOF of formate over 10 h was 1274 h^–1^ (Table S4). The lower TOF values compared to H_2_ production is attributed to FDH having a slower specific activity than H_2_ase. The ratio of [Co(bpy)_3_]^2+^ to [Co(bpy)_3_]^3+^ was found to be around 1 to 3.8 after 10 h of photocatalysis (Figure 3b,d) with a TOF for [Co(bpy)_3_]^3+/2+^ of 0.33 h^–1^. The AQY at 420 nm and the solar-to-formate energy conversion efficiency (STF) were found to be 0.5% and 0.004% (10 h), respectively.

In addition to utilizing the full simulated solar spectrum, additional experiments were conducted under UV-free visible light irradiation (λ > 420 nm). The Z-scheme H_2_ase|SrTiO_3_:La,Rh|[Co(bpy)_3_]^3+/2+^|BiVO_4_:Mo|RuO_2_ system produced 177 ± 9 nmol H_2_ in 4 h when irradiated with light >420 nm, compared to 446 ± 28 nmol H_2_ under AM 1.5G irradiation (Figure S19a). Similarly, the Z-scheme FDH|SrTiO_3_:La,Rh|[Co(bpy)_3_]^3+/2+^|BiVO_4_:Mo|RuO_2_ system generated 120 ± 16 nmol formate at >420 nm and 274 ± 22 nmol formate under AM 1.5G conditions (Figure S19b).

To evaluate the scalability of the Z-scheme system, photocatalysis experiments were conducted in a larger photoreactor, scaled up by a factor of 6 (Figure S20). Using a 6 mL Z-scheme colloidal suspension, the system produced 1890 ± 201 nmol of H_2_ and 1338 ± 189 nmol of formate in 4 h when coupled with H_2_ase and FDH, respectively (Figure S21). These values are 4.2 and 4.9 times higher than those obtained with a 1 mL colloidal suspension. The mismatch in the scale-up factor between volumes and products is attributed to the challenges in maintaining turbulent mixing in a larger stirred photoreactor, leading to partially stationary solutions. For fast turnover cocatalysts, such as molecular catalysts and enzymes, stationary solutions can cause mass transport limitations and local pH changes, resulting in reduced product yields.

To unambiguously confirm the carbon source for solar formate production, isotopic labeling experiments were conducted. Z-scheme photocatalysis experiments were carried out using either an aqueous NaH^12^CO_3_ solution (0.1 M) with ^12^CO_2_ as the headspace gas or an aqueous NaH^13^CO_3_ solution (0.1 M) with ^13^CO_2_ as the headspace gas. ^1^H NMR spectra were collected after 10 h of simulated AM 1.5G irradiation (Figure 3c), showing a singlet signal of H^12^COO^–^ (using ^12^CO_2_ and NaH^12^CO_3_) and a doublet signal of H^13^COO^–^ (using ^13^CO_2_ and NaH^13^CO_3_). ^1^H NMR spectra of commercial sodium formate-^12^C (H^12^COONa) and sodium formate-^13^C (H^13^COONa) were recorded for comparison (Figure S22). These results confirm that formate originated from CO_2_ reduction reactions, not from contaminations or side reactions. The Z-scheme SrTiO_3_:La,Rh|[Co(bpy)_3_]^3+/2+^|BiVO_4_:Mo|RuO_2_ photocatalytic system containing either H_2_ase or FDH as the biological components generated O_2_ below 0.1% volume percent within the photoreactor, which falls within the oxygen tolerance range reported for both enzymes.^23,24^

### Stability of the [Co(bpy)_3_]^3+/2+^ Redox Couple

**Figure 4:**
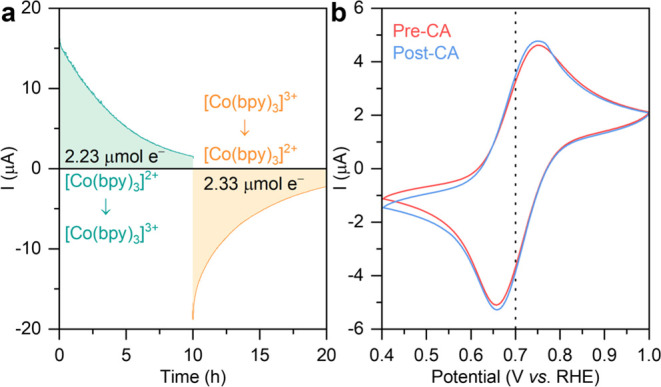
(a) Chronoamperometry of [Co(bpy)_3_]^2+^ oxidation at 0.9 V *vs* RHE and [Co(bpy)_3_]^3+^ reduction at 0.5 V *vs* RHE for 20 h. (b) Cyclic voltammetry (10 mV s^–1^, third scan) of [Co(bpy)_3_]^3+/2+^ before and after CA. The dotted line represents the redox potential. Electrolyte: a CO_2_-saturated solution (5 mL, pH 6.7) containing NaHCO_3_ (0.1 M), [Co(bpy)_3_]^2+^ (0.5 mM), and KCl (50 mM). 3-electrode configuration: Toray carbon paper working electrode (0.25 cm^2^), Ag/AgCl (saturated KCl) reference electrode, Pt mesh counter electrode.

To study the stability of the [Co(bpy)_3_]^3+/2+^ redox couple, a series of electrochemical experiments, including cyclic voltammetry (CV) and chronoamperometry (CA), were conducted. During the initial 10 h of CA (Figure 4a), [Co(bpy)_3_]^2+^ was oxidized to [Co(bpy)_3_]^3+^ at 0.9 V *vs* RHE, with the total charge passed reaching 2.23 μmol. Given that the electrochemistry was performed in a 5 mL of electrolyte containing 0.5 mM [Co(bpy)_3_]^2+^, the maximum available amount of [Co(bpy)_3_]^2+^ is 2.5 μmol, indicating the near-complete depletion of [Co(bpy)_3_]^2+^ in solution. Subsequently, the reduction of [Co(bpy)_3_]^3+^ (from the oxidized sample above) to [Co(bpy)_3_]^2+^ was conducted for another 10 h at 0.5 V *vs* RHE with a total charge pass of 2.33 μmol, representing the depletion of [Co(bpy)_3_]^3+^. The redox ability of the [Co(bpy)_3_]^3+/2+^ mediator was evaluated by CV, showing no significant deformation in CV traces (Figure 4b), indicating stability for at least 20 h. This demonstrates the suitability of the [Co(bpy)_3_]^3+/2+^ redox mediator under conditions used for the photocatalysis study presented herein.

### Characterization of Semiconductor-Enzyme Interface

**Figure 5:**
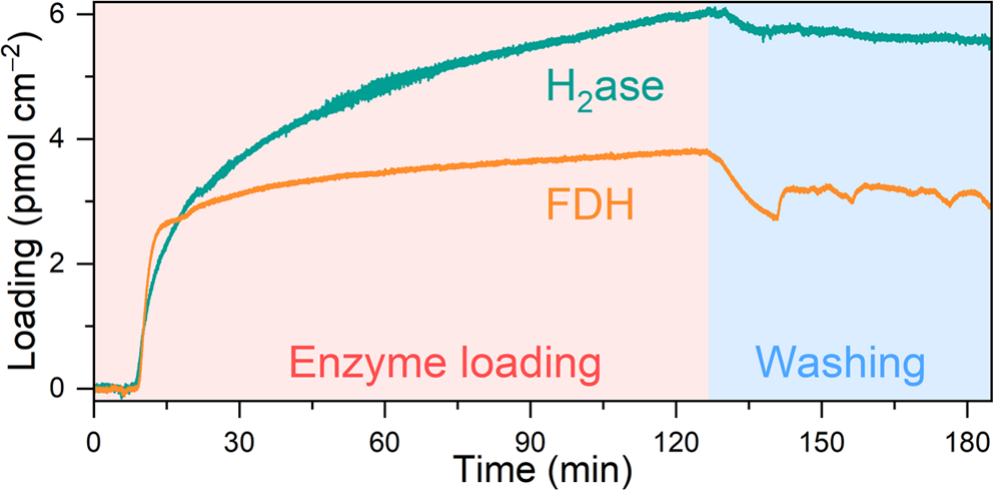
QCM analysis of the adsorption process and washing process of H_2_ase and FDH on a SrTiO_3_:La,Rh-coated quartz chip. Loading conditions: 0.141 mL min^–1^ flow rate, an anaerobic aqueous solution (2 mL, pH 6.7) containing NaHCO_3_ (0.1 M), [Co(bpy)_3_]SO_4_ (0.5 mM), and 50 pmol of enzymes (either H_2_ase or FDH), 25 °C. Washing conditions: 0.141 mL min^–1^ flow rate, an anaerobic aqueous solution (10 mL, pH 6.7) containing NaHCO_3_ (0.1 M) and 0.5 mM [Co(bpy)_3_]^3+/2+^, 25 °C.

The interactions between SrTiO_3_:La,Rh and enzymes were investigated by using QCM analysis. A gold-coated quartz chip was functionalized with a thin layer of SrTiO_3_:La,Rh by drop-casting an ultrasonicated suspension (0.1 mL) of SrTiO_3_:La,Rh (0.5 mg mL^–1^) in isopropanol, mimicking *operando* conditions during photocatalysis. As depicted in Figure 5, after establishing a stable baseline for 10 min, 50 pmol of enzymes (either H_2_ase or FDH) were introduced into an aqueous solution (2 mL) containing NaHCO_3_ (0.1 M) and [Co(bpy)_3_]SO_4_ (0.5 mM) under anaerobic conditions, with the solution flowing toward the QCM chip at a rate of 0.141 mL min^–1^. The enzyme loading was monitored and quantified by converting the frequency change into mass change using the Sauerbrey equation.^41^

The adsorption of both enzymes on SrTiO_3_:La,Rh exhibited two distinct stages: a rapid adsorption stage lasting until 22 min for H_2_ase and 13 min for FDH, followed by a slower adsorption stage until reaching an enzyme loading of 6.0 pmol cm^–2^ for H_2_ase and 3.8 pmol cm^–2^ for FDH after 2 h. Following enzyme loading, a washing process was conducted to assess the robustness of the enzyme binding with SrTiO_3_:La,Rh. An enzyme-free aqueous solution (10 mL) containing NaHCO_3_ (0.1 M) and [Co(bpy)_3_]SO_4_ (0.5 mM) was flowed at a rate of 0.141 mL min^–1^ for 1 h (Figure 5). It was observed that after enzymes were loaded onto the SrTiO_3_:La,Rh surface, their binding was strong, with only 7% of H_2_ase and 18% of FDH being desorbed after 1 h of washing.^30^ QCM studies thus confirm the robust binding between enzymes and the SrTiO_3_:La,Rh photocatalyst.

### Mechanistic Insights into Charge Carrier Dynamics

To investigate the charge carrier dynamics between the enzymes and SrTiO_3_:La,Rh, we conducted photoelectrochemical techniques, namely PEIS, TPC, and IMVS, on SrTiO_3_:La,Rh photoelectrodes. The photoelectrodes were prepared by drop-casting a SrTiO_3_:La,Rh suspension in isopropanol (50 μL of 2 mg mL^–1^) onto a masked FTO-coated glass, resulting in an active area of 0.25 cm^2^.^12^

**Figure 6:**
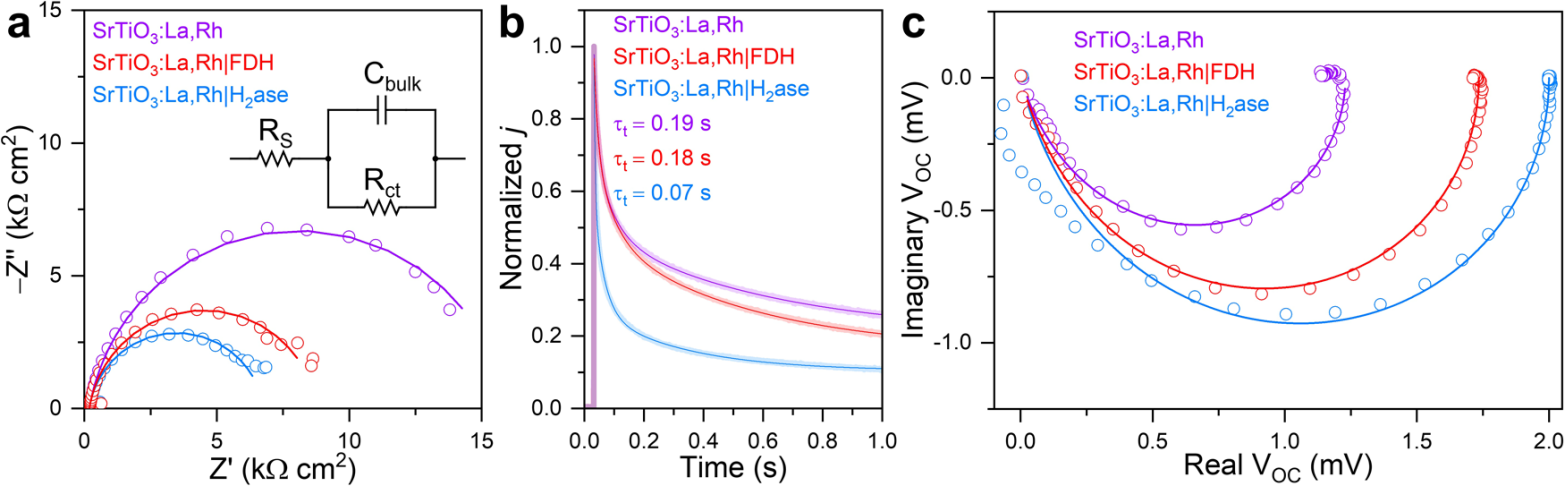
(a) Nyquist plots of the PEIS response recorded at −0.2 V *vs* RHE (open circuits) with corresponding fitted curves (solid lines). Inset: proposed equivalent circuit to fit the impedance response. (b) Normalized TPC response recorded at −0.2 V *vs* RHE with corresponding exponential fitted curves. (c) Nyquist plots of the IMVS response (open circuits) with corresponding phenomenological fitted curves (solid lines). Conditions: a CO_2_-saturated aqueous solution (20 mL, pH 6.7) containing NaHCO_3_ (0.1 M), [Co(bpy)_3_]SO_4_ (0.5 mM), and KCl (50 mM). SrTiO_3_:La,Rh, working electrode; Ag/AgCl (sat. KCl), reference electrode; Pt mesh, counter electrode; AM 1.5G irradiation; and 25 °C.

PEIS measurements were performed on pristine SrTiO_3_:La,Rh, SrTiO_3_:La,Rh|H_2_ase, and SrTiO_3_:La,Rh|FDH under simulated AM 1.5G irradiation by applying a sinusoidal voltage modulation between the working electrode and the reference electrode in an electrochemical cell with a three-electrode configuration. The impedance response displayed a single semicircle for all three samples in Figure 6a, without signs of diffusional impedance (Warburg impedance).^42^ Qualitatively, upon introducing enzymes (either H_2_ase or FDH), the semicircle’s diameter decreased, indicating a facilitated charge transfer process for Faradaic reactions.

Quantitative analysis was based on fitting the impedance response with a Randles equivalent circuit (Figure 6a inset)^43^ comprising a series resistor (*R*_S_) in series with a parallel combination of a bulk capacitor (*C*_bulk_) and a charge transfer resistor (*R*_ct_). *R*_S_ represents the sum of all non-Faradaic processes in the electrochemical system, including electrolyte resistance, contact resistance, and lead resistance. All three samples exhibited an *R*_S_ value around 188 Ω (Table S5). *R*_ct_ describes the Faradaic process of the system, specifically the difficulty of electron transfer at the electrode–electrolyte interface.^44^ The assembly between H_2_ase and SrTiO_3_:La,Rh decreased *R*_ct_ from 15.7 ± 0.16 to 6.8 ± 0.09 kΩ, indicating the role of H_2_ase as cocatalysts for catalyzing hydrogen evolution reaction (HER). Similarly, SrTiO_3_:La,Rh|FDH showed a decreased *R*_ct_ of 8.7 ± 0.09 kΩ, indicating the role of FDH in facilitating CO_2_ reduction to formate. The lower *R*_ct_ observed for SrTiO_3_:La,Rh|H_2_ase compared to SrTiO_3_:La,Rh|FDH aligns well with the difference in product yields during photocatalysis. This can be attributed to the inherently slower specific activity of FDH for CO_2_ reduction compared to H_2_ase for HER. According to the fitting results, the pseudo first-order rate constant for charge transfer (*k*_ct_) of the Faradaic process was determined based on the phenomenological model developed for photoelectrodes under irradiation.^45^ The values of *k*_ct_ for pristine SrTiO_3_:La,Rh, SrTiO_3_:La,Rh|H_2_ase, and SrTiO_3_:La,Rh|FDH were 2.6, 7.6, and 4.8 s^–1^, respectively, further confirming that enzymes can facilitate the kinetics of charge transfer processes for dedicated reactions.

In addition to investigating the reduction half reaction, PEIS was performed on BiVO_4_:Mo and BiVO_4_:Mo|RuO_2_ to study the oxidation half reaction, thereby complementing the full Z-scheme reactions (Figure S23). Randles equivalent circuit fitting reveals that the incorporation of RuO_2_ on BiVO_4_:Mo decreases the *R*_ct_ from 153.3 ± 19.44 to 79.1 ± 6.91 kΩ (Table S5). This reduction in *R*_ct_ supports the effectiveness of RuO_2_ as an efficient OER cocatalyst in Z-scheme systems.

The impact of enzymes on the electron extraction process of SrTiO_3_:La,Rh was evaluated using TPC, as depicted in Figure 6b. By employing exponential fitting on the normalized TPC response, we determined the electron transit time (τ_*t*_) values for pristine SrTiO_3_:La,Rh, SrTiO_3_:La,Rh|H_2_ase, and SrTiO_3_:La,Rh|FDH. Our findings revealed that interfacing H_2_ases with SrTiO_3_:La,Rh significantly decreased τ_*t*_ from 0.19 to 0.07 s, indicating photogenerated electrons can be effectively collected by H_2_ase for HER, thereby enhancing the electron transport process within SrTiO_3_:La,Rh. A similar trend was observed for SrTiO_3_:La,Rh|FDH, resulting in a slightly reduced τ_*t*_ value of 0.18 s.

Furthermore, IMVS was utilized to gain insights into charge recombination dynamics. This technique is well-established in solar cell research and has gained increased attention for evaluating electron lifetime in photoelectrochemical systems.^46,47^ By sinusoidally modulating incident light intensity, IMVS recorded the complex-valued open circuit photovoltage response (*V*_OC_). To accurately determine the first-order electron lifetime (τ_*n*_) of the system, phenomenological fitting of the Nyquist plot was carried out (Figure 6c):^48,49^
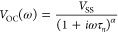
where, *V*_SS_ is steady-state photovoltage, ω is angular frequency, and α (0< α ≤ 1) is introduced as a nonideality factor to account for surface inhomogeneity and the frequency-dependent dielectric constant of SrTiO_3_:La,Rh photoelectrodes. The proposed phenomenological fitting accurately characterizes the system in Figure 6c, exhibiting a coefficient of determination near 0.99. Across all three samples, α values around 0.83 (Table S6) suggest a Cole–Cole relaxation model, where a dispersion of relaxation time constants is considered around the value of τ_*n*_.^50,51^ This model indicates deviations from an ideal Debye relaxation (α = 1), which would present a perfect semicircle in the Nyquist plot. The *V*_SS_ parameter serves as an indicator of recombination degree in an illuminated semiconductor.^52^

For pristine SrTiO_3_:La,Rh, SrTiO_3_:La,Rh|H_2_ase, and SrTiO_3_:La,Rh|FDH, *V*_*SS*_ values were found to be 1.23 ± 0.01, 2.00 ± 0.01, and 1.73 ± 0.01 mV, respectively. This indicates that both enzymes contributed to a decrease in charge recombination. Moreover, the introduction of H_2_ase and FDH onto SrTiO_3_:La,Rh reduced τ_*n*_ from 466 ± 7.0 μs to 379 ± 12.0 and 412 ± 10.0 μs, respectively. This reduction can be attributed to the efficient extraction of photogenerated charges to enzymes upon irradiation, facilitating catalytic reactions and thus decreasing τ_*n*_.

Attempts to study the charge transfer rate constant with intensity-modulated photocurrent spectroscopy (IMPS) were unsuccessful due to a low signal-to-noise ratio, which prevented the construction of a Nyquist plot. This issue is likely a result of the low photocurrent response (a few μA) of photoelectrodes made with SrTiO_3_:La,Rh nanoparticles. Given a 10% incident light modulation depth, a minimum photocurrent response of approximately 100 μA is required to generate meaningful Nyquist plots. Although SrTiO_3_ is an effective light absorber for photocatalysis, the morphology significantly impacts photocurrent generation. Unlike high-performing metal oxide photoelectrodes (*e.g.*, hematite and Cu_2_O) fabricated through hydrothermal growth or electrodeposition to achieve large crystal grain sizes, our SrTiO_3_:La,Rh photoelectrodes maintain a morphology of poorly connected nanoparticles, resulting in numerous interfaces that act as charge recombination centers.

## Conclusions

This work establishes semiartificial colloidal Z-scheme photosynthesis for the selective synthesis of solar fuels without the requirement for sacrificial reagents. The semiartificial colloidal photosynthetic Z-scheme is versatile, easy to assemble and achieved effective H_2_ production or CO_2_ reduction using water as the electron donor. The developed H_2_ase|SrTiO_3_:La,Rh|[Co(bpy)_3_]^3+/2+^|BiVO_4_:Mo|RuO_2_ system yielded 506 ± 36 μmol H_2_ g^–1^ with a TON of 50,550 and FDH|SrTiO_3_:La,Rh|[Co(bpy)_3_]^3+/2+^|BiVO_4_:Mo|RuO_2_ yielded 319 ± 27 μmol formate g^–1^ with a TON of 12,740. Mott–Schottky analysis and cyclic voltammetry confirmed an energy band alignment suitable for a Z-scheme system between SrTiO_3_:La,Rh and BiVO_4_:Mo, along with determining the formal potential of the [Co(bpy)_3_]^3+/2+^ electron mediator within their respective band gaps. QCM analysis provided insights into the rapid adsorption process and strong binding interactions of H_2_ase and FDH onto the SrTiO_3_:La,Rh surface. Furthermore, a comprehensive suite of photoelectrochemical techniques (PEIS, TPC, and IMVS) were employed to investigate charge carrier dynamics, encompassing charge transfer, transport, and recombination processes. PEIS revealed significant reductions of *R*_ct_ (57% for H_2_ase and 45% for FDH) and enhanced *k*_ct_ (192% for H_2_ase and 84% for FDH) compared to pristine SrTiO_3_:La,Rh, indicating improved charge transfer efficiency upon enzyme integration. TPC demonstrated that both enzymes facilitate the charge transport process, evidenced by a decreased τ_*t*_ upon interfacing SrTiO_3_:La,Rh with enzymes. IMVS analysis showed 19% and 12% reductions of τ_*n*_ upon introducing H_2_ase and FDH, respectively, reflecting efficient extraction of photogenerated electrons to enzymes under irradiation.

## Experimental Section

### Materials

The following chemicals and materials were purchased from commercial suppliers and used without further purification: N_2_ and CO_2_ gas bottles (2% CH_4_ as internal standard, BOC), carbon-^13^C dioxide (^13^CO_2_, Sigma-Aldrich, 99.0 atom % ^13^C), O_2_ cylinder industrial grade (99.5%, BOC), strontium carbonate (SrCO_3_, Alfa Aesar, 99.99%), rutile titanium dioxide (TiO_2_, Sigma-Aldrich, ≥99.98%), lanthanum oxide (La_2_O_3_, Fisher Scientific, 99.99%), rhodium(III) oxide (Rh_2_O_3_, Wako Pure Chemical, 98.0–102.0%), molybdenum trioxide (MoO_3_, BDH Chemicals, 99.5%), vanadium(V) oxide (V_2_O_5_, Fisher Scientific, 99.6%), bismuth(III) nitrate pentahydrate (Bi(NO_3_)_3_·5H_2_O, Sigma-Aldrich, 98%), ruthenium(III) chloride hydrate (RuCl_3_·*x*H_2_O, Acros Organics, 35–40% Ru), cobalt(II) sulfate heptahydrate (CoSO_4_·7H_2_O, Acros Organics, 99+%), 2,2′-bipyridine (C_10_H_8_N_2_, Alfa Aesar, 98%), DL-dithiothreitol (DTT, Sigma-Aldrich, >99.5%), 2-amino-2-(hydroxymethyl)-1,3-propanediol (Tris base, Sigma-Aldrich, ≥99.8%), hydrochloric acid (HCl, Honeywell Fluka, 37%), sodium bicarbonate (NaHCO_3_, Sigma-Aldrich, ≥99.9%), sodium bicarbonate-^13^C (NaH^13^CO_3_, Sigma-Aldrich, 98 atom % ^13^C), sodium formate (HCOONa, Sigma-Aldrich, ≥ 99.0%), sodium formate-^13^C (H^13^COONa, Sigma-Aldrich, 99 atom % ^13^C), isopropanol ((CH_3_)_2_CHOH, Sigma-Aldrich, ≥99.5%), methanol (CH_3_OH, anhydrous, Sigma-Aldrich, 99.8%), ethanol (C_2_H_5_OH, Sigma-Aldrich, 96%), deuterium oxide (D_2_O, Sigma-Aldrich, 99.9 atom % D, contains 0.75 wt % 3-(trimethylsilyl)propionic-2,2,3,3-d_4_ acid, sodium salt), Nafion perfluorinated resin solution (Sigma-Aldrich, 5 wt % in mixture of lower aliphatic alcohols and water, contains 45% water), Toray carbon paper (TGP-H-60, Thermo Scientific Chemicals), fluorine doped tin oxide (FTO) coated glass slide (2 cm × 10 cm, Pilkington TEC 15, Xop Glass, 12–14 Ω/sq), Parafilm (Bemis), and rubber septa (Subaseal). Milli-Q H_2_O (18.2 MΩ cm) was used for all of the experiments. [W]-FDH and [NiFeSe]-H_2_ase from *D. vulgaris* Hildenborough (*Dv*H) were expressed and purified according to previously reported methods.^23,24^

### Synthesis of La,Rh Codoped SrTiO_3_

La,Rh codoped strontium titanate, denoted as SrTiO_3_:La,Rh, was synthesized by a two-step, solid-state reaction based on a previously reported procedure.^12,13^ SrCO_3_ was preheated at 573 K in air for 1 h before mixing with TiO_2_ in a mortar at a Sr/Ti molar ratio of 1.05. The mixture was heated to 1473 K (10 K min^–1^) for 10 h in an alumina crucible. The resulting SrTiO_3_ was cooled down naturally to room temperature before mixing with La_2_O_3_ at a La/(La + Sr) ratio of 4 mol % and Rh_2_O_3_ at a Rh/(Rh + Ti) ratio of 4 mol %. The mixture was heated to 1373 K for 6 h to obtain SrTiO_3_:La,Rh with La/(La + Sr)=Rh/(Rh + Ti) = 4 mol %.

### Synthesis of RuO_2_-Loaded Mo-Doped BiVO_4_

Mo-doped bismuth vanadate, denoted as BiVO_4_:Mo (Mo/V = 0.05 mol %), was prepared following literature.^53^ K_2_CO_3_, MoO_3_, and V_2_O_5_ (molar ratio of 1.05:0.05:1) were calcined in air at 723 K for 5 h to form a layered Mo-doped K_3_V_5_O_14_ precursor. A suspension of BiONO_3_ was prepared by adding stoichiometric Bi(NO_3_)_3_·5H_2_O to distilled water. The Mo-doped K_3_V_5_O_14_ was added to this suspension, and the mixture was stirred at 343 K for 10 h. The resulting BiVO_4_:Mo was collected by filtration and washed with distilled water. RuO_2_ cocatalyst (1 wt %) was deposited onto BiVO_4_:Mo *via* impregnation.^13^ 100 mg of BiVO_4_:Mo and 751.3 μL of 0.01 M RuCl_3_ solution were added to an evaporating dish. The mixture was briefly sonicated to disperse the BiVO_4_:Mo. The mixture was stirred continuously while being evaporated over a water bath. The obtained powder was calcined in air at 623 K for 1 h.

### Synthesis of [Co(bpy)_3_]SO_4_

Degassed water (15 mL) was added under vacuum to a Schlenk tube containing 2,2′-bipyridine (bpy, 258 mg, 1.65 mmol) and Co(SO_4_)·7H_2_O (139 mg, 0.49 mmol) and the suspension was stirred under N_2_ for 10 min.^14,34^ Subsequently, dry methanol (15 mL) was added under low pressure and continuous stirring. The Schlenk tube was then evacuated and purged with N_2_ for 15 min before the orange-yellow solution was heated to 90 °C for 2 h. After cooling down the reaction, it was taken to dryness and the mustard-colored solid was suspended in chloroform (20 mL), briefly sonicated and filtered off. The resulting mustard-colored solid was washed with chloroform (2 mL × 15 mL) and diethyl ether (2 mL × 15 mL) and dried under vacuum overnight to yield a mustard crystalline powder (300 mg, 81%, Figure S15). The reaction scheme is shown in Figure S14. Elemental analysis; calc. for C_30_H_24_N_6_O_4_SCo·7.05H_2_O (*M* = 750.56 g mol^–1^): C: 48.01, H 5.12, N 11.20%. Found: C 48.07, H 4.65, N 10.73%. ^1^H NMR (D_2_O, 400 MHz): δ (ppm) = 87.99 (bs, 6H_a_), 83.46 (s, 6H_d_), 45.91 (s, 6H_c_), 14.52 (s, 6H_b_). ESI-MS (+, water): *m*/*z* calc. for C_30_H_24_N_6_Co^2+^ (*i.e.*, M^2+^): 263.5692, found: 263.5704; calc. for C_30_H_24_N_6_Co^+^ (*i.e.*, M^+^): 527.1383, found: 527.1507; calc. for C_30_H_25_N_6_Co^+^ (*i.e.*, [M + H^+^]^+^): 528.1462, found: 528.1493. UV–vis (50 mM KCl, 100 mM NaHCO_3_ in H_2_O): λ_max_ (nm) (ε, M^–1^ cm^–1^) = 435 (101). ATR-FTIR: v (cm^–1^) = 3229, 3076, 1672, 1597, 1470, 1440, 1312, 1055, 1019, 775, 737.

### Physical Characterizations

SEM images were acquired on a TESCAN MIRA3 field emission-gun-scanning electron microscope (FEG-SEM). TEM images were acquired on a Thermo Scientific (FEI) Talos F200X G2 TEM. EDX spectroscopy was carried out on the TESCAN MIRA3 FEG-SEM equipped with an Oxford Instruments Aztec Energy X-Max^N^ 80 mm^2^ silicon drift detector. XPS data were acquired on a Thermo Scientific Escalab 250Xi fitted with a monochromated aluminum Kα X-ray source (1486.7 eV) at a pressure below 10–8 Torr and a room temperature of 294 K. XRD patterns were measured with a Malvern Panalytical Empyrean Series 2 X-ray diffractometer using Cu Kα irradiation operated at a 40 kV generator voltage and 40 mA tube current. Elemental analysis was carried out by using a PerkinElmer 240 elemental analyzer. High-resolution mass spectra were recorded on an Agilent 1260 Infinity LC system coupled to an Agilent 6230 time-of-flight liquid chromatography–mass spectrometry (LC/MS) system. ^1^H NMR spectra were collected with a 500 MHz Avance III Smart Probe NMR spectrometer at room temperature. Chemical shifts for ^1^H NMR spectra are referenced relative to residual protons in the deuterated solvent (Eurisotop), and 3-(trimethylsilyl)propionic-2,2,3,3-d_4_ acid, sodium salt, in D_2_O was used as the internal standard (TSP). ATR-FTIR spectra were recorded on a Nicolet iS50 spectrometer. UV–vis spectra were collected using a Cary 60 UV–vis spectrometer.

### Photocatalysis

Photoreactor information and lamp setup can be found in Figures S24 and S25. For Z-scheme half reactions, either SrTiO_3_:La,Rh (1 mg) or BiVO_4_:Mo|RuO_2_ (1 mg) was ultrasonicated for 30 min and dispersed in an aqueous solution (1 mL) containing NaHCO_3_ (0.1 M) and [Co(bpy)_3_]^3+/2+^ (0.5 mM). For SrTiO_3_:La,Rh experiments, either H_2_ase (20 pmol) or FDH (50 pmol) was added anaerobically. For Z-scheme reactions, SrTiO_3_:La,Rh (1 mg) was ultrasonicated for 30 min and dispersed in an aqueous solution (0.5 mL) containing NaHCO_3_ (0.1 M) and [Co(bpy)_3_]SO_4_ (0.5 mM). The SrTiO_3_:La,Rh suspension was first anaerobically mixed with either H_2_ase (20 pmol) or FDH (50 pmol) in the dark for 30 min, then mixed with BiVO_4_:Mo|RuO_2_ (1 mg in 0.5 mL) suspension containing NaHCO_3_ (0.1 M) and [Co(bpy)_3_]SO_4_ (0.5 mM). All of the photoreactors were assembled in an anaerobic glovebox (MBraun, N_2_ atmosphere, <0.1 ppm of O_2_) and purged with CO_2_ for 10 min. During photocatalysis, the photoreactors were illuminated under simulated AM 1.5G (100 mW cm^–2^, Newport xenon arc lamp housing 66,921) and stirred at 600 rpm at 25 °C. For visible light-driven photocatalysis, a 420 nm long-pass filter was positioned in front of the photoreactors. Prior semiconductor–enzyme assembly, FDH was incubated with DTT solution (80 mM) in Tris-HCl buffer (20 mM, pH 9) for 10 min to activate the enzymes.^24^

### Product Quantification

The amount of H_2_ produced was analyzed by headspace gas analysis using a Shimadzu Tracera GC-2010 Plus with a barrier discharge ionization detector. The GC-2010 Plus was equipped with a ShinCarbon micro ST column (0.53 mm diameter) kept at 40 °C using helium carrier gas. Aliquots of the headspace gas (100 μL) were removed from the sealed photocatalytic reactors using a gastight syringe (Hamilton) for GC analysis. CH_4_ was used as an internal standard. The amount of formate produced was quantified by ion chromatography (IC) using a Metrohm 882 Compact IC Plus ion chromatograph with a conductivity detector and a pump pressure of around 10 MPa. The eluent buffer contained Na_2_CO_3_ (3 mM) and NaHCO_3_ (1 mM) in H_2_O. Aliquots of the photocatalysis solution were removed from the sealed photocatalytic reactors and diluted 10 times with H_2_O before injecting into the ion chromatograph *via* a 220 nm syringe filter. O_2_ was quantified by a NeoFox-GT fluorometer and Fospor-R fluorescence oxygen sensor probe (Ocean Optics) in a glovebox (Belle Technology, N_2_ atmosphere, <1 ppm of O_2_).

### Determination of [Co(bpy)_3_]^3+/2+^ Concentrations

The quantification method is based on the work of Mulazzani et al.^36^ and Kudo et al.^14^ using the Beer–Lambert law: *A* = ε*cl*, where *A* is the absorbance, ε is the molar absorption coefficient (M^–1^ cm^–1^), *c* is the molar concentration (*M*), and *l* is the optical path length (cm).

### Isotopic Labeling

Photocatalysis experiments were carried out in either a NaH^12^CO_3_ (0.1 M) aqueous solution with ^12^CO_2_ as the headspace gas or a NaH^13^CO_3_ (0.1 M) aqueous solution with ^13^CO_2_ as the headspace gas. After 10 h of simulated AM 1.5G irradiation, the solution was transferred to an NMR tube, and ^1^H NMR spectra were collected with a 400 MHz NMR spectrometer. ^1^H NMR spectra of commercial sodium formate-^12^C (H^12^COONa) and sodium formate-^13^C (H^13^COONa) were recorded to compare with the ^1^H NMR spectra of the labeled products (Figure S22).

### Apparent Quantum Yield (AQY)

AQY measurements were conducted on a solar simulator (LOT-Quantum Design , LSN254) with a monochromator (LOT-Quantum Design, MSH300) at a 420 nm wavelength. The incident light was measured by a power meter (Thorlabs, PM100D) with a thermal power sensor (Thorlabs, S302C). The calculation of AQY for the Z-scheme reactions is based on the two-step photoexcitation mechanism: the production of one H_2_ or formate molecule requires the generation of two photoexcited electrons by SrTiO_3_:La,Rh, and another two photoexcited electrons by BiVO_4_:Mo|RuO_2_ to recombine with the two holes in SrTiO_3_:La,Rh *via* the redox mediator. Therefore, one produced H_2_ or formate molecule requires four photoexcited electrons. The AQY is calculated by
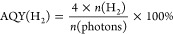

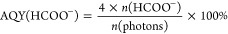
where, *n*(H_2_), n(HCOO^–^), and n(photons) represent the number of produced H_2_, number of produced formate, and number of incident photons, respectively.

### Solar Energy Conversion Efficiency

The solar-to-H_2_ energy conversion efficiency (STH) is described as
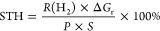
where, *R*(H_2_), Δ*G*_r_, *P*, and *S* are the rate of H_2_ evolution, the reaction Gibbs energy of the water splitting (237.2 kJ mol^–1^), the AM 1.5G irradiance (100 mW cm^–2^), and the irradiated sample area (1 cm^2^), respectively.

Similarly, the solar-to-formate energy conversion efficiency (STF) is given by
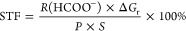
where, *R*(HCOO^–^) and Δ*G*_r_ are the rate of formate evolution and the reaction Gibbs energy of the CO_2_-to-formate conversion (238 kJ mol^–1^), respectively.

### Quartz Crystal Microbalance (QCM)

QCM experiments were performed using a Biolin Q-Sense Explorer module and a custom-designed QCM cell within an anaerobic glovebox (MBraun, N_2_ atmosphere, <0.1 ppm of O_2_). A gold-coated quartz chip with a surface area of 0.79 cm^2^ and a surface roughness <1 nm RMS was utilized. The chip was initially functionalized by drop-casting an ultrasonicated suspension (0.1 mL) of SrTiO_3_:La,Rh (0.5 mg mL^–1^) in isopropyl alcohol, forming a thin layer on the surface. To establish a stable baseline, prior to measurements, an enzyme-free aqueous solution (2 mL) containing NaHCO_3_ (0.1 M) and [Co(bpy)_3_]SO_4_ (0.5 mM) was flowed through the system at a rate of 0.141 mL min^–1^ for a duration of at least 1 h. Once the baseline reached a steady state, 50 pmol of enzyme (either H_2_ase or FDH) was introduced into the buffer solution (2 mL) for evaluating enzyme loading for 2 h. Subsequently after enzyme loading, a washing process was performed by flowing an enzyme-free aqueous solution (10 mL) containing NaHCO_3_ (0.1 M) and [Co(bpy)_3_]SO_4_ (0.5 mM) at a rate of 0.141 mL min^–1^ for a duration of 1 h. The adsorption and desorption of the enzyme onto the surface was quantified by monitoring changes in the resonance frequency of the piezoelectric quartz chip. To determine the corresponding mass change, the change in frequency (Δ*f*) was analyzed using the Sauerbrey equation:^41^
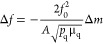
where, *f*_0_ is the resonance frequency (5 MHz) of the quartz oscillator, *A* is the piezoelectrically active crystal area, Δ*m* is the change in mass, *p*_q_ is the density of quartz, and μ_q_ is the shear modulus of quartz. Assuming 25% of the adsorbed mass consisted of water molecules bound to the enzymes, Δ*m* can be converted into the quantity of enzymes.

### Electrode Fabrication

The thin-film electrodes were made by depositing nanoparticle suspension in isopropanol on a FTO-coated glass, adapting a literature procedure.^12,13^ A 0.25 cm^2^ Parafilm template, made with a drilling bill, was pressed onto the FTO side (1 cm × 2 cm) and slightly heated (10 s in a 150 °C drying oven) to ensure uniform adhesion of the mask to the slide. The suspensions of SrTiO_3_:La,Rh, BiVO_4_:Mo, or BiVO_4_:Mo|RuO_2_ were dispersed in isopropanol at a concentration of 10 mg mL^–1^ and ultrasonicated for 2 min. In total, 50 μL of the suspension was drop-cast onto the masked FTO glass in 4 equal layers (12.5 μL per layer) and allowed to dry in air. For SrTiO_3_:La,Rh and BiVO_4_:Mo|RuO_2_ electrodes, the mask was removed and the samples were annealed for 1 h at 573 K in air (ramp rate 5 °C min^–1^). For BiVO_4_:Mo electrodes, 2 μL of Nafion solution was diluted in 50 μL of isopropanol then drop-cast onto BiVO_4_:Mo and allowed to dry in air. Photographs of these thin-film electrodes demonstrate that the metal oxide semiconductors have been homogeneously deposited onto a well-defined area (0.25 cm^2^), ensuring high quality and uniformity of the thin-film electrodes (Figure S26).

### Electrochemical Impedance Spectroscopy (EIS)

EIS experiments were performed in an electrochemical cell with a three-electrode configuration: a working electrode (either SrTiO_3_:La,Rh or BiVO_4_:Mo), a Pt mesh counter electrode, and a RE-6 Ag/AgCl reference electrode (3 M NaCl gel, 0.55 mm diameter ceramic frit, MW-2030, BASi). 50 pmol of enzymes (either H_2_ase or FDH) was drop-cast onto the working electrode. The anaerobic electrolyte (20 mL) contains CO_2_-saturated NaHCO_3_ (0.1 M), pH 6.7, KCl (50 mM), and [Co(bpy)_3_]SO_4_ (0.5 mM). Photoelectrochemical impedance spectroscopy (PEIS) measurements were carried out under AM 1.5G irradiation where a 150 W xenon arc lamp (LOT-Quantum Design , LSE140/160.25C) was used as a light source. Impedance response was recorded at −0.2 V *vs* RHE with a potentiostat (IviumStat) with frequency ranges from 100 kHz to 50 mHz and a 25 mV sinusoidal amplitude. Impedance data was fitted with equivalent circuits using modeling software ZView2 (Scribner Associates).

### Transient Photocurrent Spectroscopy (TPC)

TPC measurements were conducted in a single compartment electrochemical cell with a three-electrode configuration containing a SrTiO_3_:La,Rh working electrode, a Pt mesh counter electrode, and a RE-6 Ag/AgCl reference electrode (3 M NaCl gel, 0.55 mm diameter ceramic frit, MW-2030, BASi). 50 pmol of enzymes (either H_2_ase or FDH) were drop-cast onto the working electrode. The anaerobic electrolyte (20 mL) contains CO_2_-saturated NaHCO_3_ (0.1 M), pH 6.7, KCl (50 mM), and [Co(bpy)_3_]SO_4_ (0.5 mM). An AM 1.5G solar light simulator (LOT-Quantum Design, LS0816-H/LSN558) with a built-in shutter was used as the light source. TPC response was recorded at −0.2 V *vs* RHE on a Bio-Logic VSP potentiostat. TPC data were normalized and fitted with exponential decay function using OriginPro 2021b (OriginLab).

### Intensity-Modulated Photovoltage Spectroscopy (IMVS)

IMVS measurements were carried out under open circuit conditions in a single compartment electrochemical cell with a two-electrode configuration containing a SrTiO_3_:La,Rh working electrode and a RE-6 Ag/AgCl reference electrode (3 M NaCl gel, 0.55 mm diameter ceramic frit, MW-2030, BASi). 50 pmol of enzymes (either H_2_ase or FDH) were drop-cast onto the working electrode. The anaerobic electrolyte (20 mL) contains CO_2_-saturated NaHCO_3_ (0.1 M), pH 6.7, KCl (50 mM), and [Co(bpy)_3_]SO_4_ (0.5 mM). A 470 nm blue LED (TruOpto, OSUB5111P, 5 mm, 12000 mcd) was used as the light source and was sinusoidally modulated (0.5 MHz to 0.5 Hz, ∼10% modulation depth) by a Bio-Logic VSP potentiostat. The open circuit voltage (*V*_OC_) was recorded on a Bio-Logic VSP potentiostat.

## Data Availability

Data supporting the findings of this study are available from the University of Cambridge data repository: https://doi.org/10.17863/CAM.112500.

## Supporting Information Available

Tauc plots; Mott–Schottky plots; SEM images; TEM images; EDX mapping; XRD patterns; UV–vis spectra; ^1^H NMR spectra; ATR-FTIR spectrum; photoreactor information; lamp setup; photograph of electrodes (PDF)
