# Supplementary material for: Solar Fuel Synthesis Using a Semiartificial Colloidal Z-Scheme
Source: J Am Chem Soc. 2024 Oct 16;146(43):29865–76. doi: 10.1021/jacs.4c11827 (PMC11528412; doi:10.1021/jacs.4c11827)
Supplement: Supplementary file 1 — ja4c11827_si_001.pdf [file ja4c11827_si_001.pdf]

Supporting Information

Solar Fuel Synthesis Using a Semiartificial Colloidal Z-Scheme

Yongpeng Liu,[1,§] Ariffin Bin Mohamad Annuar,[1,§] Santiago Rodríguez-Jiménez,[1] Celine Wing See Yeung,[1] Qian Wang,[1,#] Ana M. Coito,[2] Rita R. Manuel,[2] Inês A. C. Pereira,[2] Erwin Reisner[1,*]

[1]Yusuf Hamied Department of Chemistry, University of Cambridge, Cambridge CB2 1EW, U.K.

[2]Instituto de Tecnologia Química e Biológica António Xavier (ITQB NOVA), Universidade NOVA de Lisboa, 2780-157 Oeiras, Portugal

[§]Y.L. and A.B.M.A contributed equally to this work

[#]Present Address: Graduate School of Engineering, Nagoya University, Furo-cho, Chikusa-ku, Nagoya 464-8603, Japan

E-mail: reisner@ch.cam.ac.uk

Contents

S1 Tauc Plots of $SrTiO_3$:La,Rh and $BiVO_4$:Mo . . . S7

S2 Mott−Schottky Analysis of $SrTiO_3$:La,Rh and $BiVO_4$:Mo . . . S8

S3 Scanning Electron Microscopy (SEM) Images . . . S10

S3.1 SEM Images for $SrTiO_3$:La,Rh . . . S10

S3.2 SEM Images for $BiVO_4$:Mo . . . S11

S3.3 SEM Images for $BiVO_4$:Mo|RuO_2 . . . S11

S4 Transmission Electron Microscopy (TEM) Images . . . S12

S4.1 TEM Images for $SrTiO_3$:La,Rh . . . S12

S4.2 TEM Images for $BiVO_4$:Mo . . . S13

S4.3 TEM Images for $BiVO_4$:Mo|RuO_2 . . . S13

S5 Energy-Dispersive X-ray (EDX) Mapping . . . S14

S5.1 EDX Mapping for $SrTiO_3$:La,Rh . . . S14

S5.2 EDX Mapping for $BiVO_4$:Mo . . . S15

S5.3 EDX Mapping for $BiVO_4$:Mo|RuO_2 . . . S16

S6 Doping-Induced Powder X-ray Diffraction (XRD) Peaks Shift . . . S17

S7 UV−Vis Spectra of $SrTiO_3$:La,Rh and $BiVO_4$:Mo . . . S18

S8 Reaction Scheme of $[Co(bpy)_3]SO_4$ . . . S19

S9 Photograph of $[Co(bpy)_3]SO_4$ . . . S19

S10 ^{1}H NMR Spectrum of $[Co(bpy)_3]SO_4$ . . . S20

S11 ATR-FTIR Spectrum of $[Co(bpy)_3]SO_4$ . . . S21

S12 UV−Vis Spectrum of $[Co(bpy)_3]SO_4$ . . . S22

S13 Table of Z-Scheme H_2 Evolution Half Reactions . . . S23

S14 Table of Z-Scheme CO_2 Reduction Half Reactions . . . S23

S15 Table of Z-Scheme Reactions . . . S23

S16 Comparison among State-of-the-art $SrTiO_3$−$BiVO_4$ Z-Scheme Photocatalysts for Solar H_2 Evolution and CO_2 Reduction . . . S24

S17 Z-Scheme Photocatalysis under Visible Light S25
S18 Large Photoreactor and Closure Information S26
S19 Z-Scheme Photocatalysis with Large Photoreactors S27
S20 Isotopic Labeling Experiments S28
S21 Table of PEIS Fitting Results S29
S22 Table of IMVS Fitting Results S29
S23 Impedance Analysis on the Oxidation Half Reaction S30
S24 Small Photoreactor and Closure Information S31
S25 Light Source Setup S32
S26 Photograph of Electrodes S33
References S34

List of Figures

S1 Tauc plots of $SrTiO_3$:La,Rh and $BiVO_4$:Mo S7

S2 Mott–Schottky plots of $SrTiO_3$:La,Rh and $BiVO_4$:Mo S9

S3 SEM images of $SrTiO_3$:La,Rh S10

S4 SEM images of $BiVO_4$:Mo S11

S5 SEM images of $BiVO_4$:Mo|RuO_2 . . . . S11

S6 TEM images for $SrTiO_3$:La,Rh S12

S7 TEM images for $BiVO_4$:Mo S13

S8 TEM images for $BiVO_4$:Mo|RuO_2 . . . . S13

S9 EDX mapping for $SrTiO_3$:La,Rh S14

S10 EDX mapping for $BiVO_4$:Mo S15

S11 EDX mapping for $BiVO_4$:Mo|RuO_2 . . . . S16

S12 Doping-induced XRD peaks shift S17

S13 UV−vis spectra of $SrTiO_3$:La,Rh and $BiVO_4$:Mo S18

S14 Reaction scheme of cobalt(II) tris(bipyridine) sulphate S19

S15 Photograph of cobalt(II) tris(bipyridine) sulphate S19

S16 ^{1}H NMR spectrum of $Co(bpy)_3(SO_4)$ S20

S17 ATR-FTIR spectrum of $Co(bpy)_3(SO_4)$ S21

S18 UV−vis spectrum of $Co(bpy)_3(SO_4)$ S22

S19 Z-scheme photocatalysis under visible light S25

S20 Photograph of the closure process of large photoreactors and specifications of the photoreactor and rubber septa S26

S21 Z-scheme photocatalysis with large photoreactors S27

S22 Isotopic labeling experiments S28

S23 Impedance analysis on the oxidation half reaction S30

S24 Photograph of the closure process of small photoreactors and specifications of the photoreactor and rubber septa S31

S25 Photograph of the light source setup S32

S26 Photograph of electrodes S33

List of Tables

S1 Table of Z-scheme H_2 evolution half reactions S23

S2 Table of Z-scheme CO_2 reduction half reactions S23

S3 Table of Z-scheme reactions S23

S4 Comparison among state-of-the-art $SrTiO_3-BiVO_4$ Z-scheme photocatalysts for solar H_2 Evolution and CO_2 reduction S24

S5 Table of PEIS fitting results S29

S6 Table of IMVS fitting results S29

S1 Tauc Plots of $SrTiO_3$:La,Rh and $BiVO_4$:Mo

To evaluate the electronic band structures of $SrTiO_3$:La,Rh and $BiVO_4$:Mo, we conducted Tauc plot analysis derived from UV–vis spectra (Figure S13)[S1] and Mott–Schottky analysis from electrochemical impedance spectroscopy (EIS) to determine the optical band gap and conduction band edge, respectively. The Tauc plot (Figure S1a) revealed that the indirect band gap semiconductor $SrTiO_3$:La,Rh has a band gap energy of 2.98 eV. On the other hand, as shown in Figure S1b, the direct band gap semiconductor $BiVO_4$:Mo has a band gap energy of 2.43 eV.

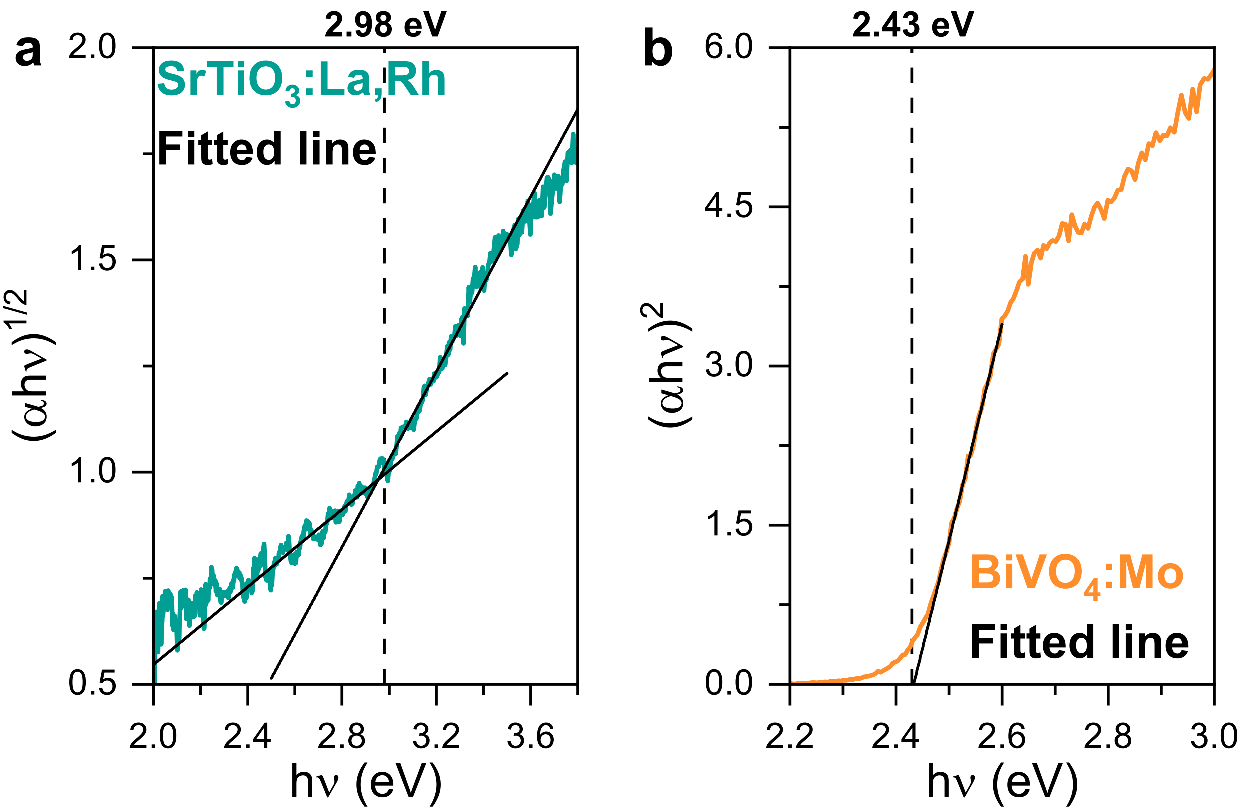

Figure S1: Tauc plots and corresponding fitted lines for (a) $SrTiO_3$:La,Rh and (b) $BiVO_4$:Mo.

S2 Mott–Schottky Analysis of $SrTiO_3$:La,Rh and $BiVO_4$:Mo

To gain further insights into the band edge positions, Mott–Schottky analysis is carried out:[S2]

$$C_{SC}^{-2} = \frac{2}{q\varepsilon\varepsilon_0 A^2 N_D}(V - V_{fb} - \frac{kT}{q})$$

where q is elementary charge, ε is relative permittivity (300 for $SrTiO_3$ and 50 for $BiVO_4$),[S3,S4] ε_0 is vacuum permittivity, A is effective surface area (corrected for surface roughness),[S2] N_D is donor density, V_{fb} is flat-band potential, k is Boltzmann constant and T is absolute temperature.

The Mott–Schottky plots for both $SrTiO_3$:La,Rh (Figure S2c) and $BiVO_4$:Mo (Figure S2d) exhibit linear regions with positive slopes, confirming their n-type semiconducting properties. Linear regression analysis of these regions yields a V_{fb} of –0.526 V vs RHE for $SrTiO_3$:La,Rh and 0.137 V vs RHE for $BiVO_4$:Mo. Likewise, the values of N_D were found to be 1.23×10^{18} cm^{-3} and 2.37×10^{18} cm^{-3} for $SrTiO_3$:La,Rh and $BiVO_4$:Mo, respectively. Assuming the density-of-state effective mass for electrons (m_{de}) of doped $SrTiO_3$ and doped $BiVO_4$ are 5.5×10^{-30} kg and 2.7×10^{-31} kg,[S5,S6] respectively. The effective density of states in the conduction band (N_C) is given by:[S7]

$$N_C \equiv 2(\frac{2\pi m_{de} kT}{h^2})^{\frac{3}{2}}$$

where h is Planck constant.

The value of N_C were determined to be 3.65×10^{20} cm^{-3} for $SrTiO_3$:La,Rh and 4.08×10^{18} cm^{-3} for $BiVO_4$:Mo. Given that N_D/N_C <0.05, $SrTiO_3$:La,Rh is categorized as a nondegenerate semiconductor. The Boltzmann statistics are applicable in this context:[S7]

$$E_C - E_F = kT\ln(\frac{N_C}{N_D})$$

where E_C is conduction band edge and E_F is Fermi level energy.

Conversely, as N_D/N_C >0.05, $BiVO_4$:Mo is considered a degenerate semiconductor. In such scenarios where E_F is close to E_C, the value of Fermi–Dirac integral is necessary to estimate E_F:[S7]

$$E_F - E_C = kT[\ln(\frac{N_D}{N_C}) + 2^{-\frac{3}{2}}(\frac{N_D}{N_C})]$$

At flat-band conditions, the Fermi position was observed to be 146 mV below E_C for $SrTiO_3$:La,Rh and 8.7 mV below E_C for $BiVO_4$:Mo. Consequently, the values of E_C were determined to be –0.672 V vs RHE for $SrTiO_3$:La,Rh and 0.128 V vs RHE for $BiVO_4$:Mo. Combining this information with the optical band gap values derived from the Tauc plots, the valence band edges (E_V) were estimated to be 2.31 V vs RHE and 2.56 V vs RHE for $SrTiO_3$:La,Rh and $BiVO_4$:Mo, respectively.

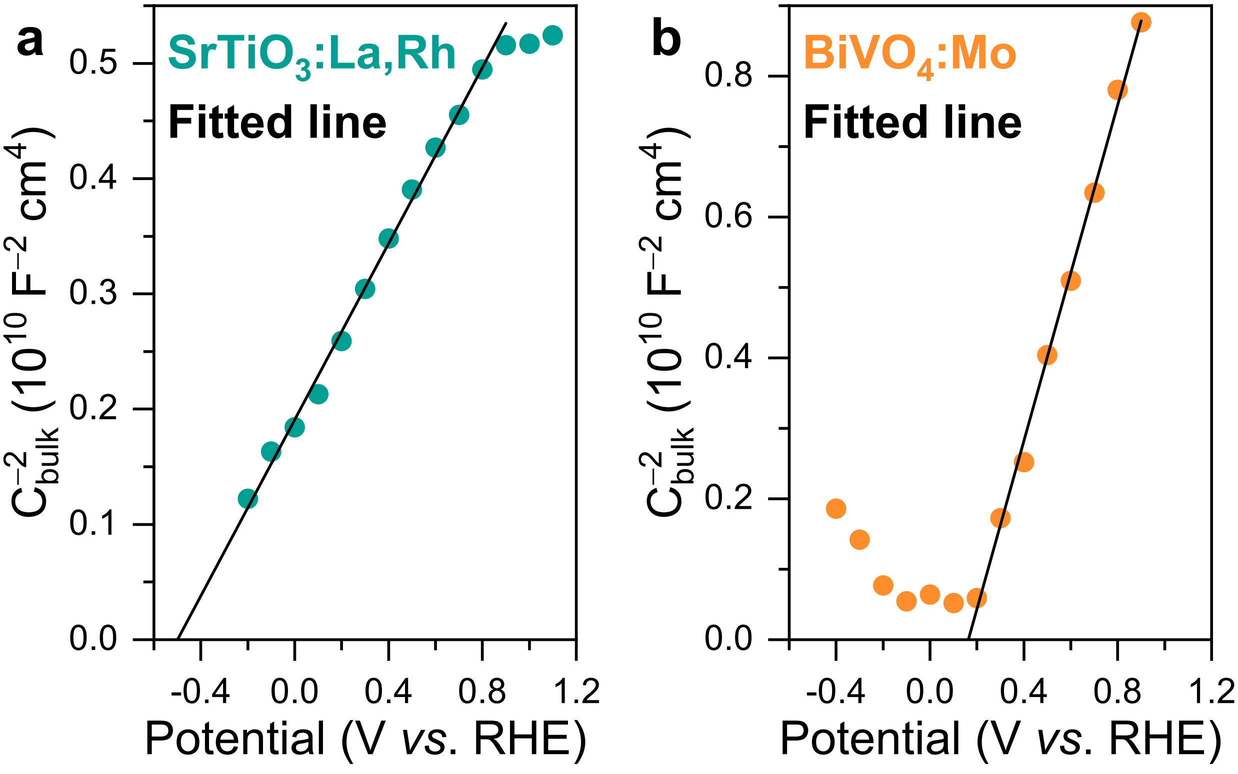

Figure S2: Mott–Schottky plots and corresponding fitted lines for (a) $SrTiO_3$:La,Rh and (b) $BiVO_4$:Mo.

S3 Scanning Electron Microscopy (SEM) Images

S3.1 SEM Images for $SrTiO_3$:La,Rh

SEM images in Figure S3 show nanoparticle morphology of $SrTiO_3$:La,Rh.

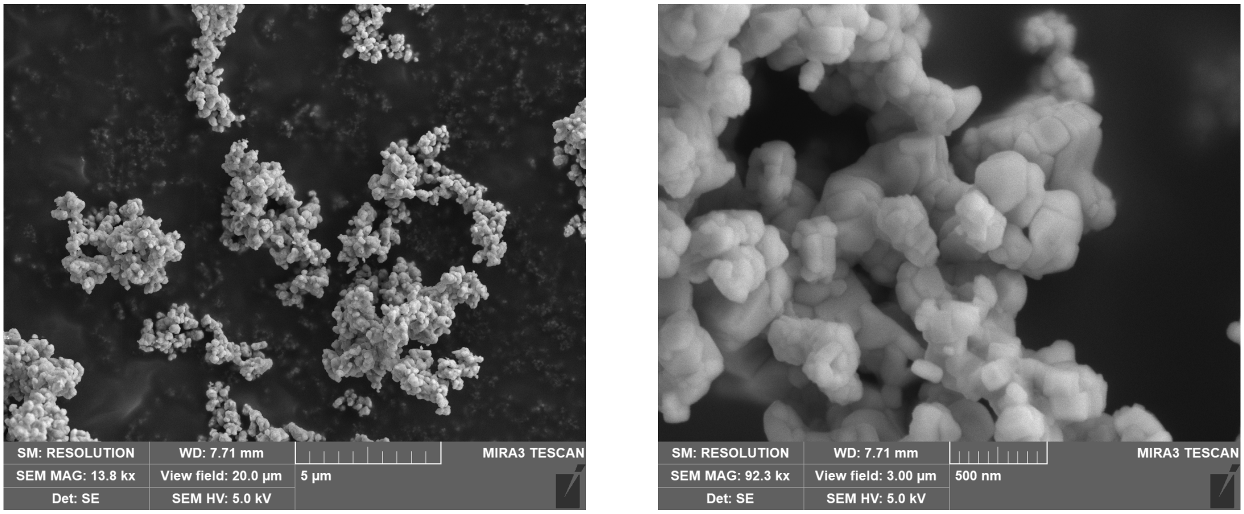

Figure S3: SEM images of $SrTiO_3$:La,Rh.

S3.2 SEM Images for $BiVO_4$:Mo

SEM images in Figure S4 show nanoplate morphology of $BiVO_4$:Mo.

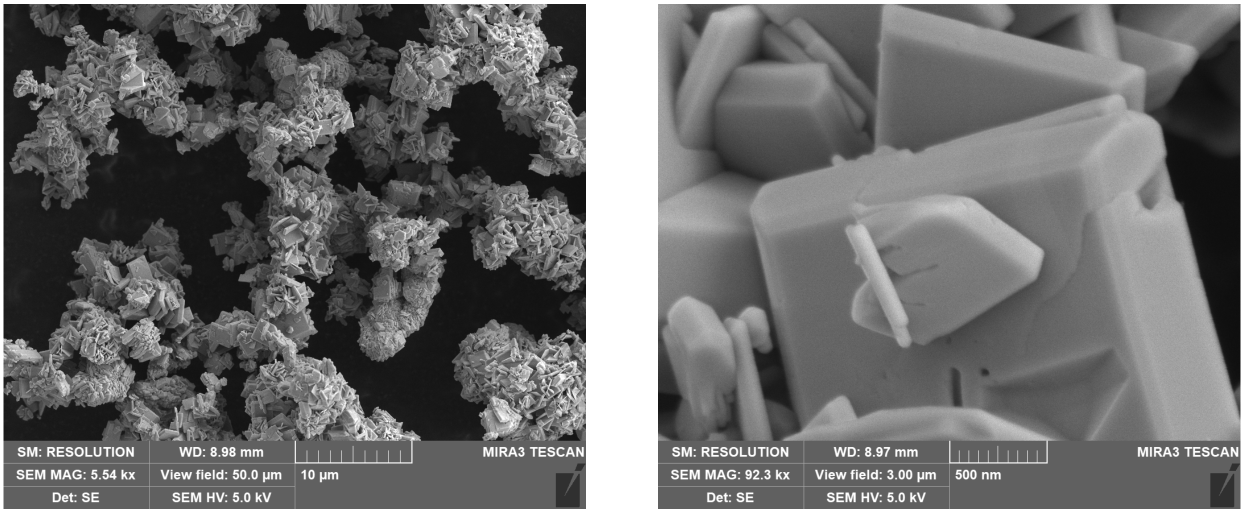

Figure S4: SEM images of $BiVO_4$:Mo.

S3.3 SEM Images for $BiVO_4$:Mo|RuO_2

SEM images in Figure S5 show the loading of RuO_2 nanoparticles on $BiVO_4$:Mo nanoplates.

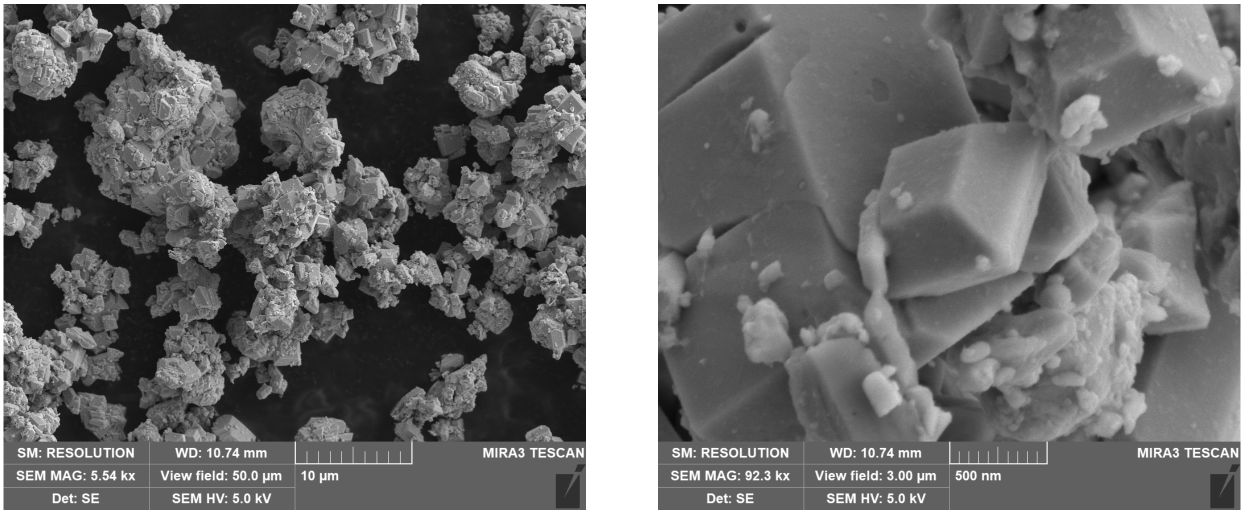

Figure S5: SEM images of $BiVO_4$:Mo|RuO_2.

S4 Transmission Electron Microscopy (TEM) Images

S4.1 TEM Images for $SrTiO_3$:La,Rh

TEM images in Figure S6 show nanoparticle morphology of $SrTiO_3$:La,Rh.

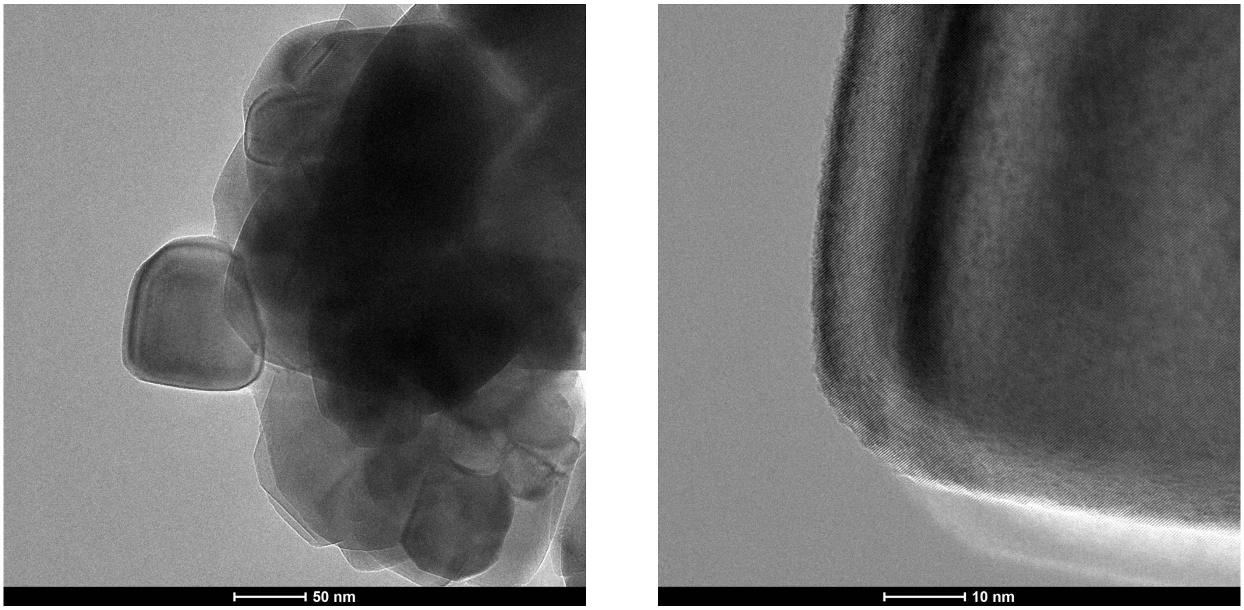

Figure S6: TEM images for $SrTiO_3$:La,Rh.

S4.2 TEM Images for $BiVO_4$:Mo

TEM images in Figure S7 show nanoplate morphology of $BiVO_4$:Mo.

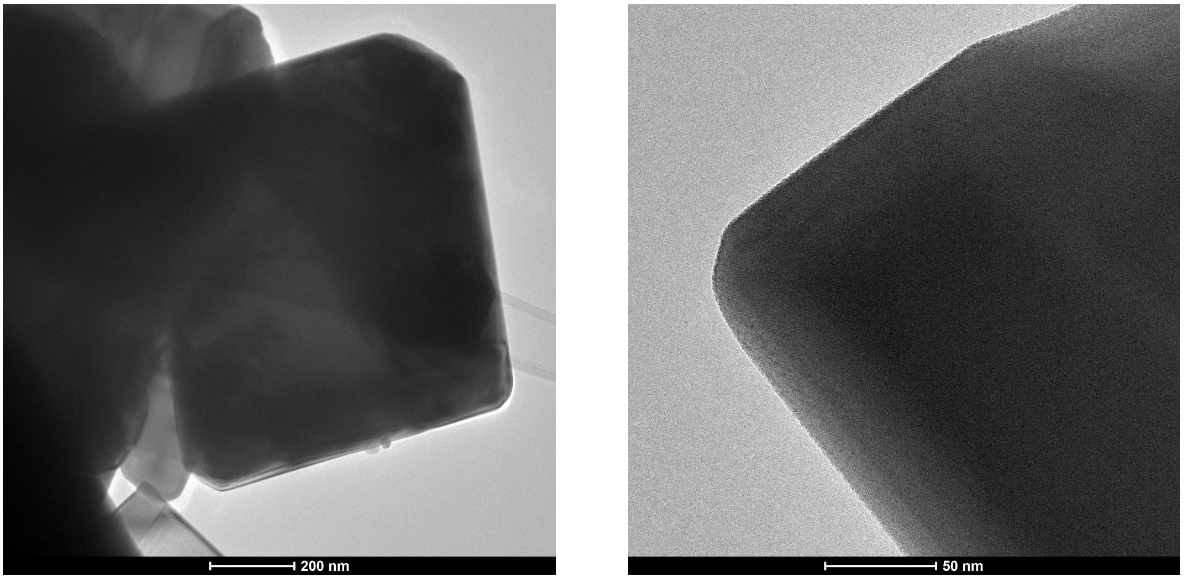

Figure S7: TEM images for $BiVO_4$:Mo.

S4.3 TEM Images for $BiVO_4$:Mo|RuO_2

TEM images in Figure S8 show the loading of RuO_2 nanoparticles on $BiVO_4$:Mo nanoplates.

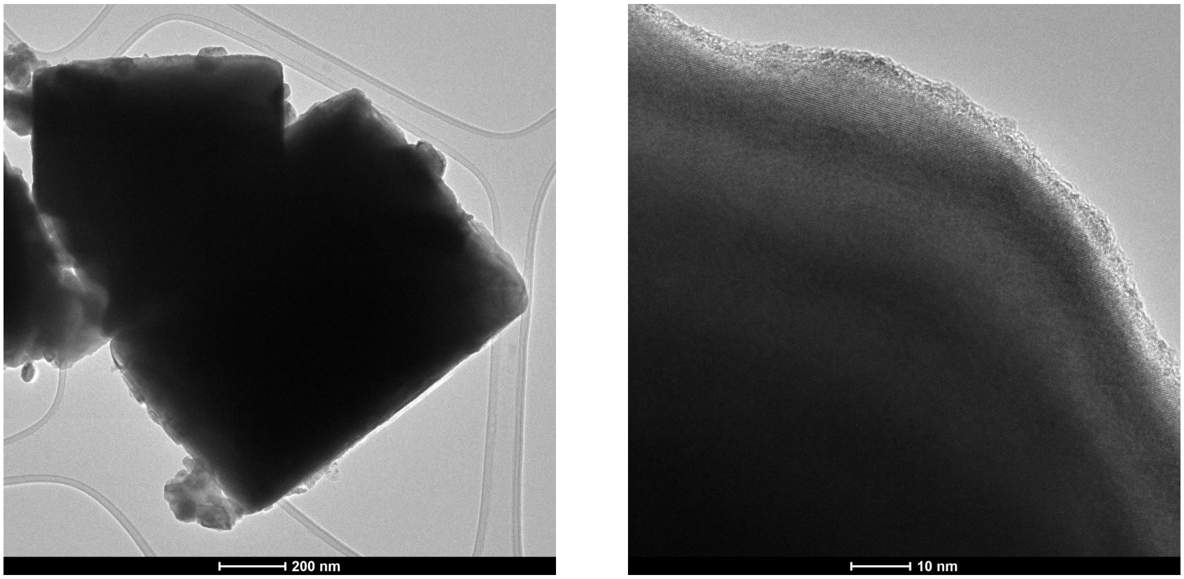

Figure S8: TEM images for $BiVO_4$:Mo|RuO_2.

S5 Energy-Dispersive X-ray (EDX) Mapping

S5.1 EDX Mapping for $SrTiO_3$:La,Rh

Figure S9 shows EDX mapping for $SrTiO_3$:La,Rh where Sr, Ti, O, La, and Rh elements are clearly identified.

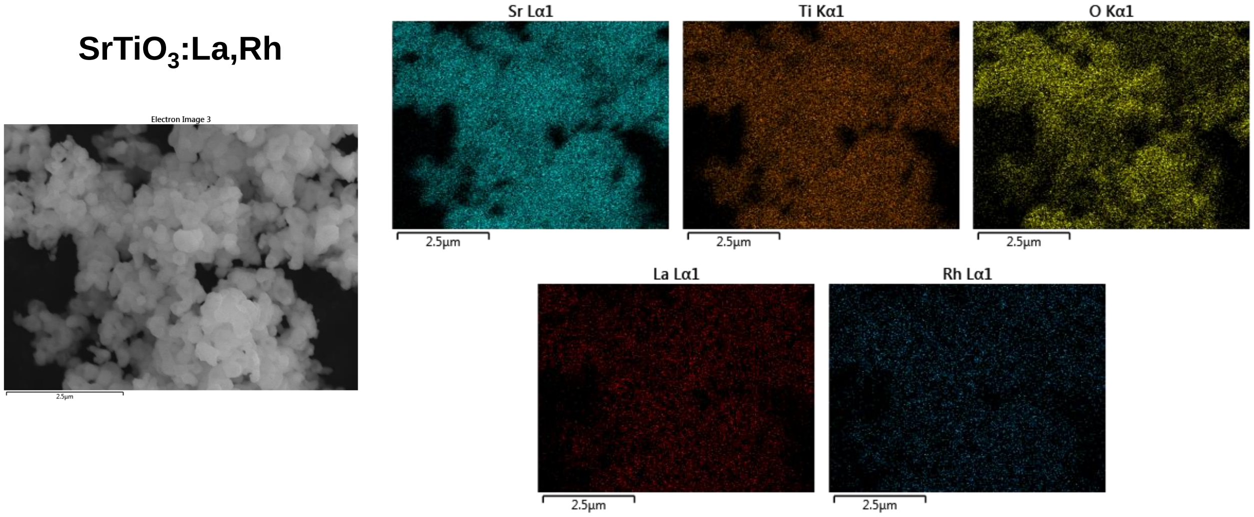

Figure S9: EDX mapping for $SrTiO_3$:La,Rh.

S5.2 EDX Mapping for $BiVO_4$:Mo

Figure S10 shows EDX mapping for $BiVO_4$:Mo where Bi, V, O, and Mo elements are clearly identified.

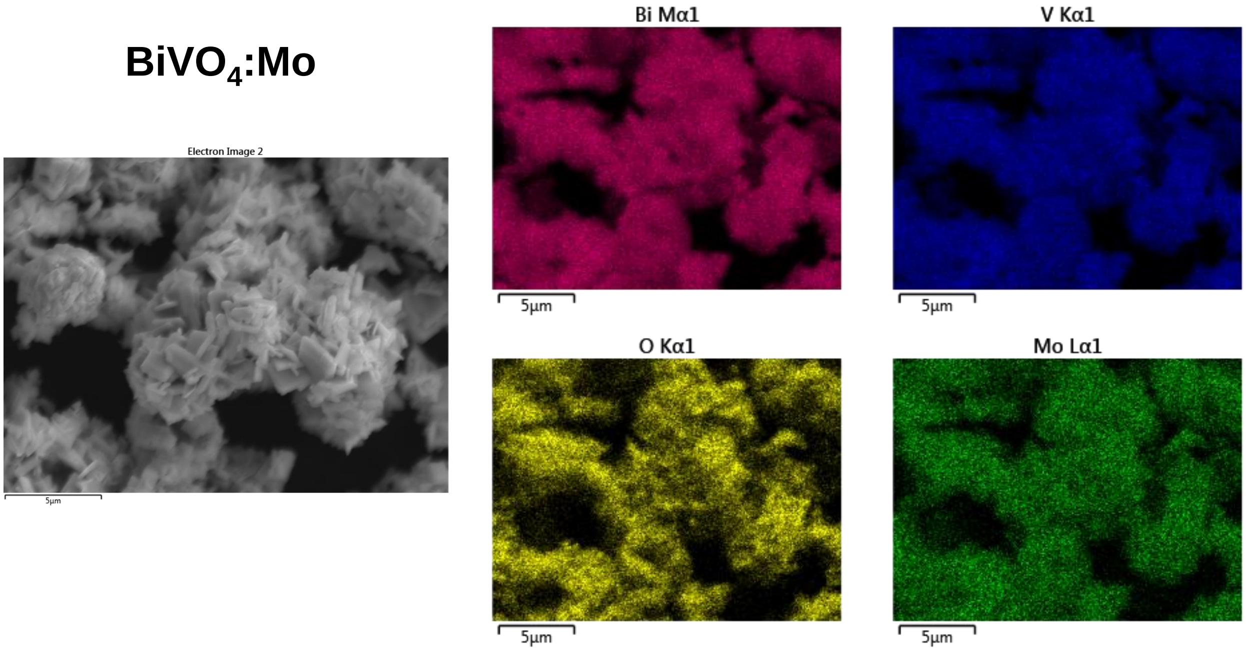

Figure S10: EDX mapping for $BiVO_4$:Mo.

S5.3 EDX Mapping for $BiVO_4$:Mo|RuO_2

Figure S11 shows EDX mapping for $BiVO_4$:Mo|RuO_2 where Bi, V, and O elements are clearly identified. Note that the proximity of the principal characteristic line energies between Mo (Lα = 2.2932 eV) and Ru (Lα = 2.5586 eV) complicates the deconvolution of elemental mapping, potentially resulting in overlapping signals when mapping either Mo or Ru. To unambiguously confirm the presence of each element, we conducted XPS analysis, as shown in Figure 1d–e in the main article.

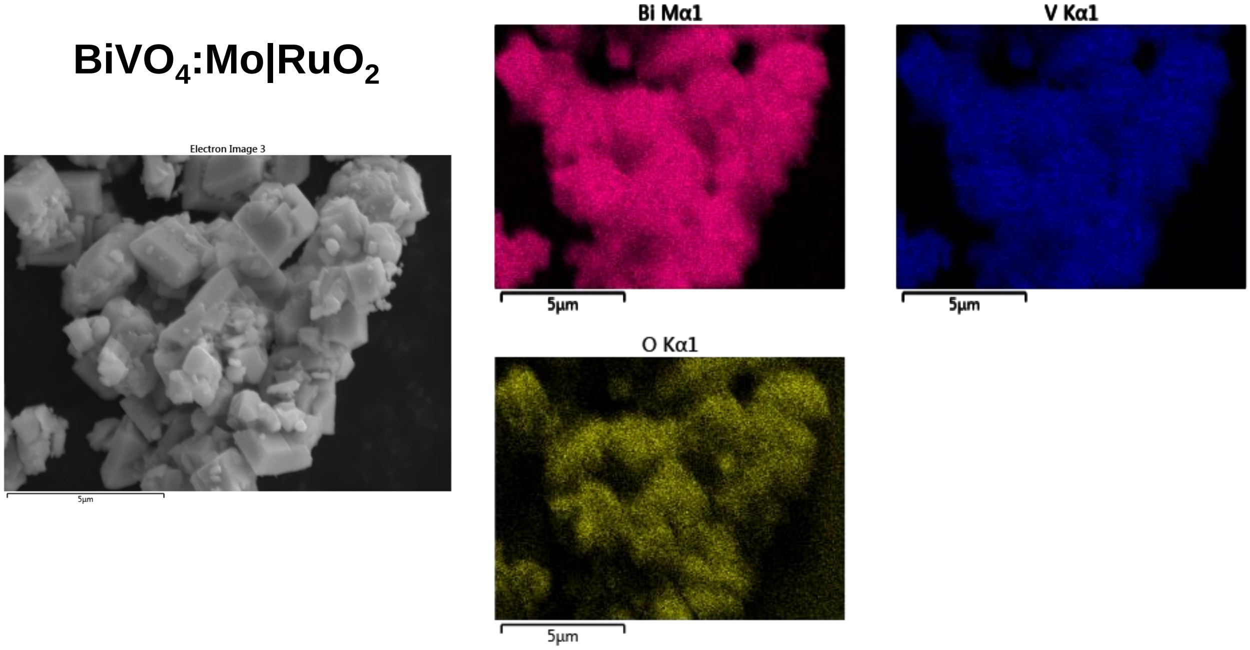

Figure S11: EDX mapping for $BiVO_4$:Mo|RuO_2.

S6 Doping-Induced Powder X-ray Diffraction (XRD) Peaks Shift

Doping-induced XRD peaks shift for $SrTiO_3$:La,Rh (Figure S12a) and $BiVO_4$:Mo (Figure S12b).

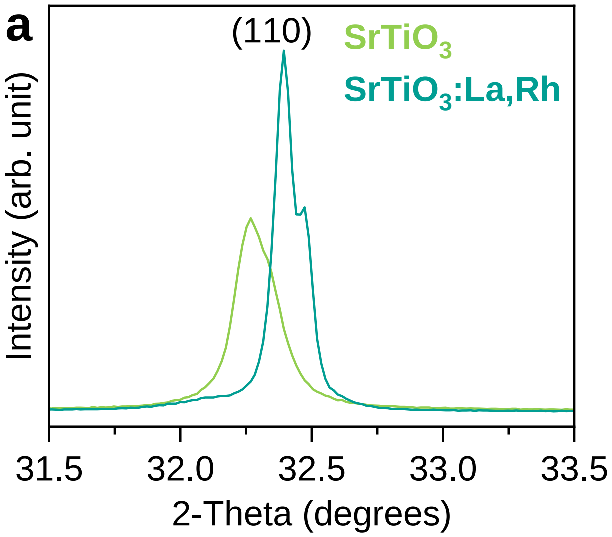

Figure S12: Doping-induced XRD peaks shift for (a) $SrTiO_3$:La,Rh and (b) $BiVO_4$:Mo.

S7 UV−Vis Spectra of $SrTiO_3$:La,Rh and $BiVO_4$:Mo

UV−vis spectra in Figure S13 show an onset wavelength (λ_{onset}) around 450 nm for $SrTiO_3$:La,Rh and a λ_{onset} around 650 nm for $BiVO_4$:Mo. Further Tauc plot analysis reveals a band gap of 2.98 eV (416 nm) for $SrTiO_3$:La,Rh and 2.43 eV (510 nm) for $BiVO_4$:Mo (Figure S1). Note that La and Rh co-doping introduced sub-bandgap states which enables visible light absorption.[S8]

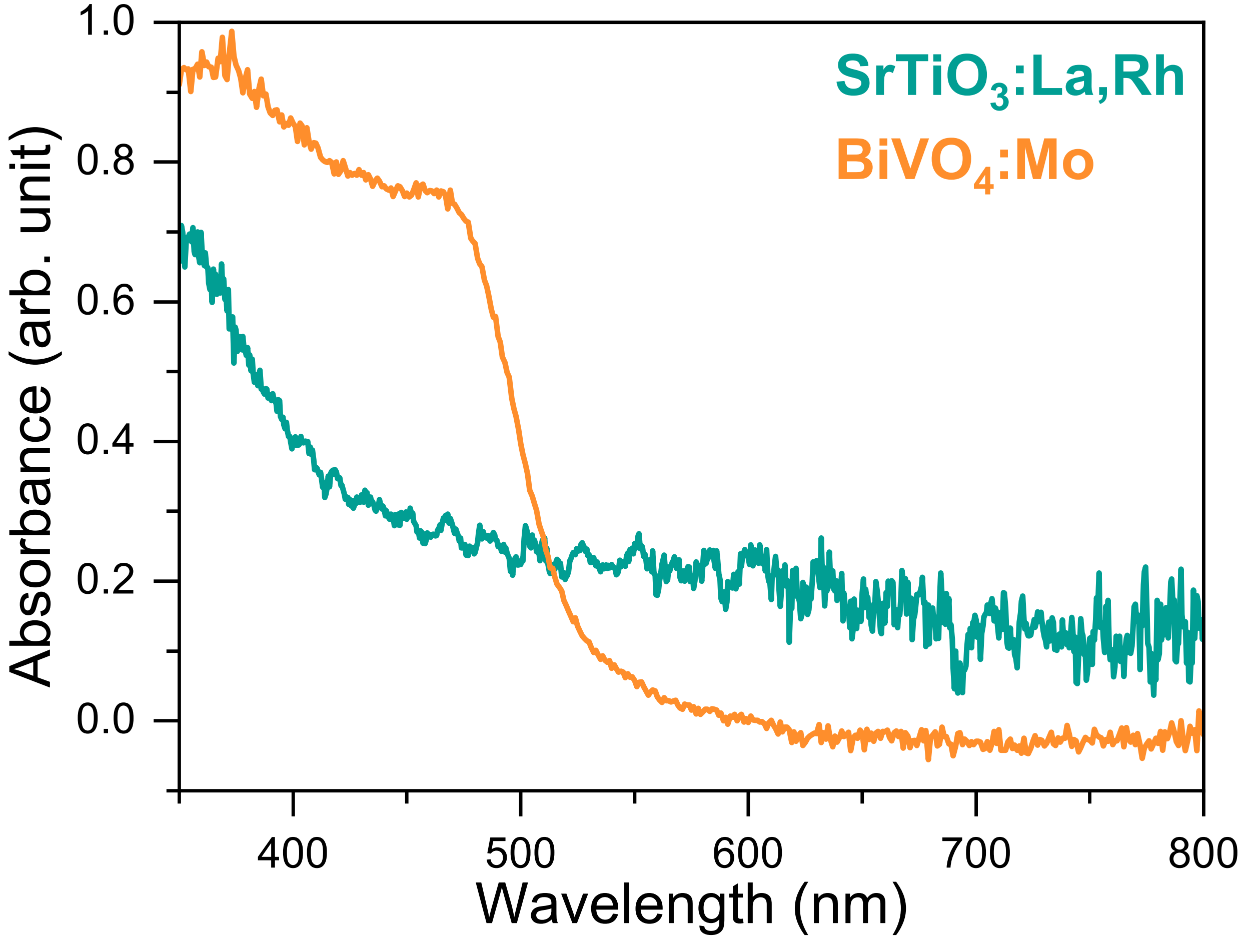

Figure S13: UV−vis spectra of $SrTiO_3$:La,Rh and $BiVO_4$:Mo.

S8 Reaction Scheme of $[Co(bpy)_3]SO_4$

The reaction scheme of $[Co(bpy)_3]SO_4$ is shown in Figure S14.

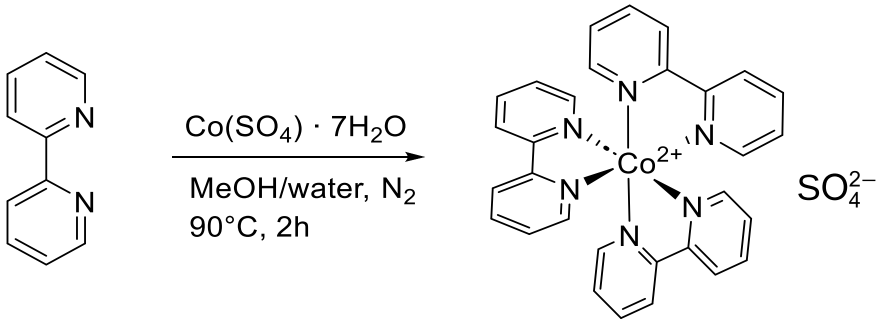

Figure S14: Reaction scheme of cobalt(II) tris(bipyridine) sulphate, $[Co(bpy)_3]SO_4$.

S9 Photograph of $[Co(bpy)_3]SO_4$

As shown in the photograph in Figure S15, the colour of $[Co(bpy)_3]SO_4$ powder is yellow, same as the previously reported colour for $[Co(bpy)_3]^{2+}$.[S9,S10]

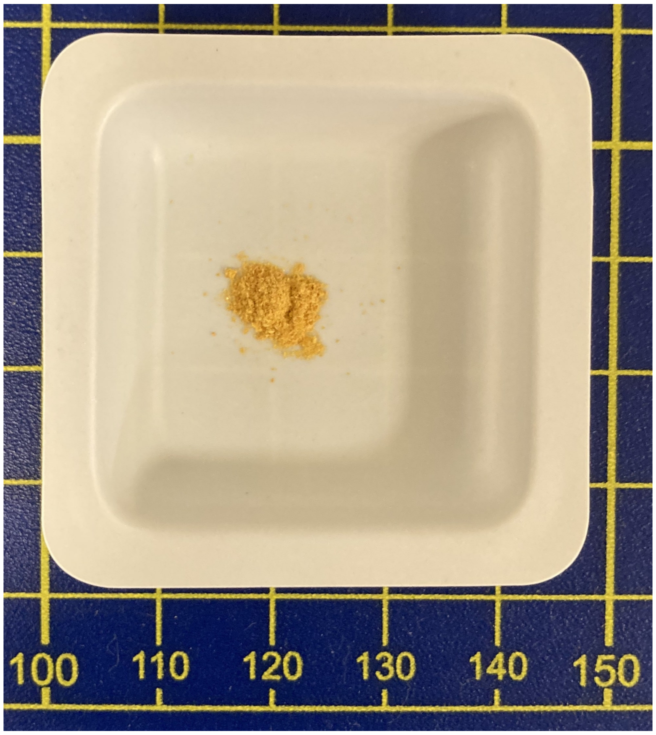

Figure S15: Photograph of $[Co(bpy)_3]SO_4$. Grid unit: mm.

S10 ^{1}H NMR Spectrum of $[Co(bpy)_3]SO_4$

The ^{1}H NMR spectrum of cobalt(II) tris(2,2'-bipyridine) sulfate complex in D_2O shows an expected paramagnetic response (Figure S16). This shift is analogous to that seen in other cobalt(II) tris-bipyridine complexes[S11] and confirms the oxidation state of the paramagnetic cobalt(II) species in solution. Note that to ensure that cobalt(II) tris(2,2'-bipyridine) sulfate remained unoxidized during the NMR measurements, we prepared the aqueous solution under anaerobic conditions in a J. Young NMR tube inside a glovebox.

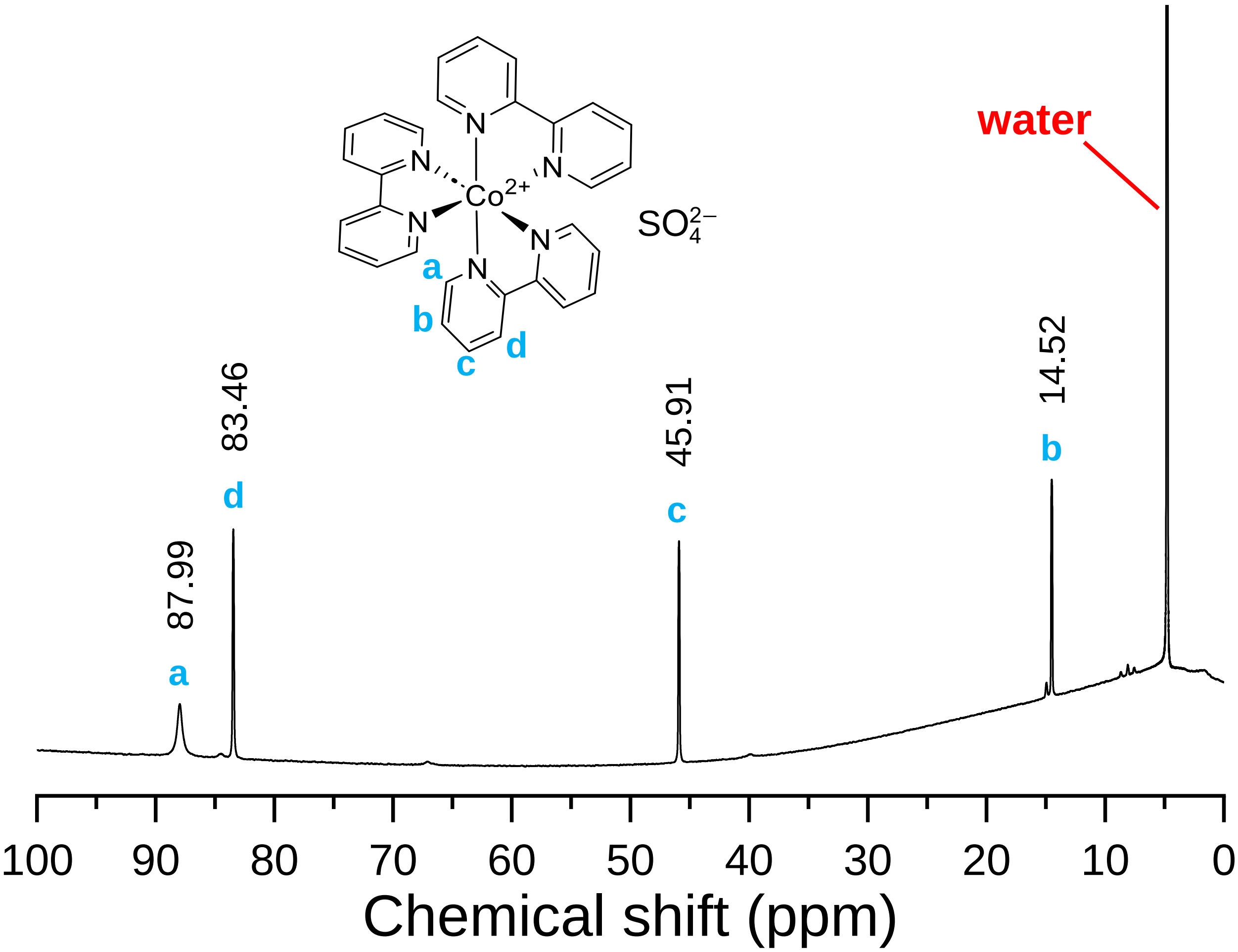

Figure S16: ^{1}H NMR spectrum of $[Co(bpy)_3]SO_4$ (1 mM) in D_2O under N_2.

S11 ATR-FTIR Spectrum of $[Co(bpy)_3]SO_4$

ATR-FTIR spectrum of cobalt(II) tris(2,2'-bipyridine) sulfate complex (Figure S17) shows characteristic bands of 2,2'-bipyridine, including C-N stretching and C-C stretching in the range of 1600 and 1300 cm^{-1}.[S12,S13]

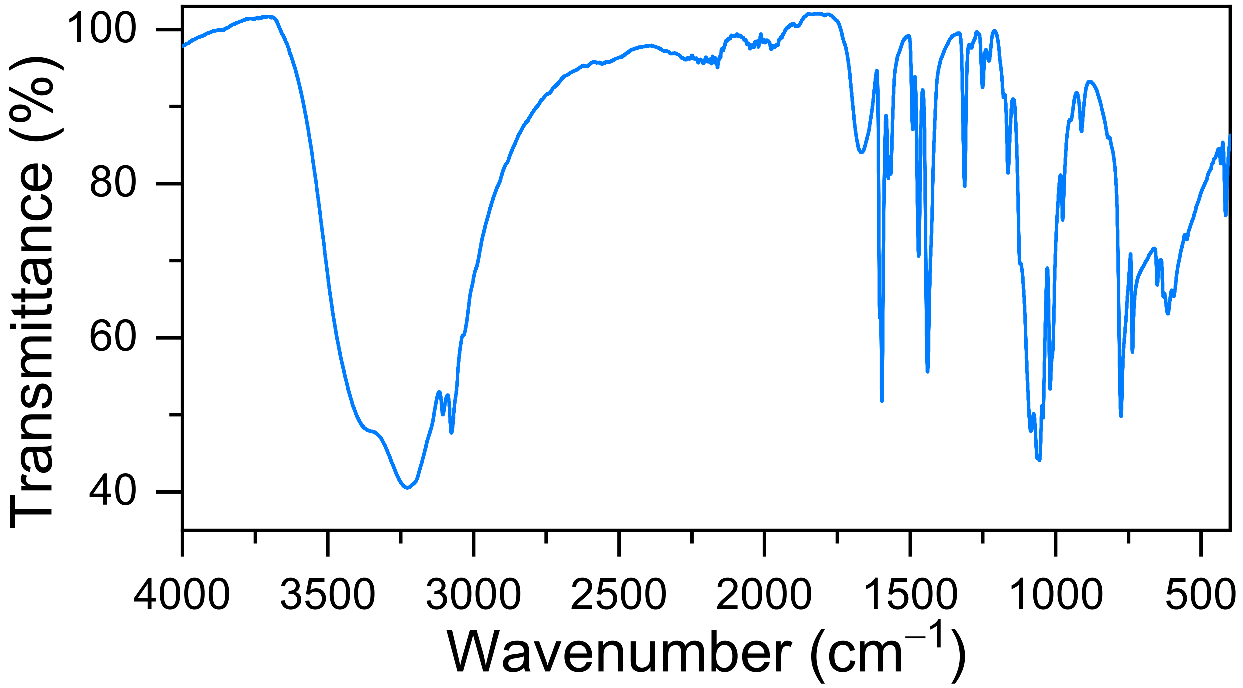

Figure S17: ATR-FTIR spectrum of $[Co(bpy)_3]SO_4$ powder.

S12 UV−Vis Spectrum of $[Co(bpy)_3]SO_4$

UV−vis spectrum of $[Co(bpy)_3]SO_4$ in the range of 350 nm to 600 nm (Figure S18) shows an absorption onset around 550 nm.

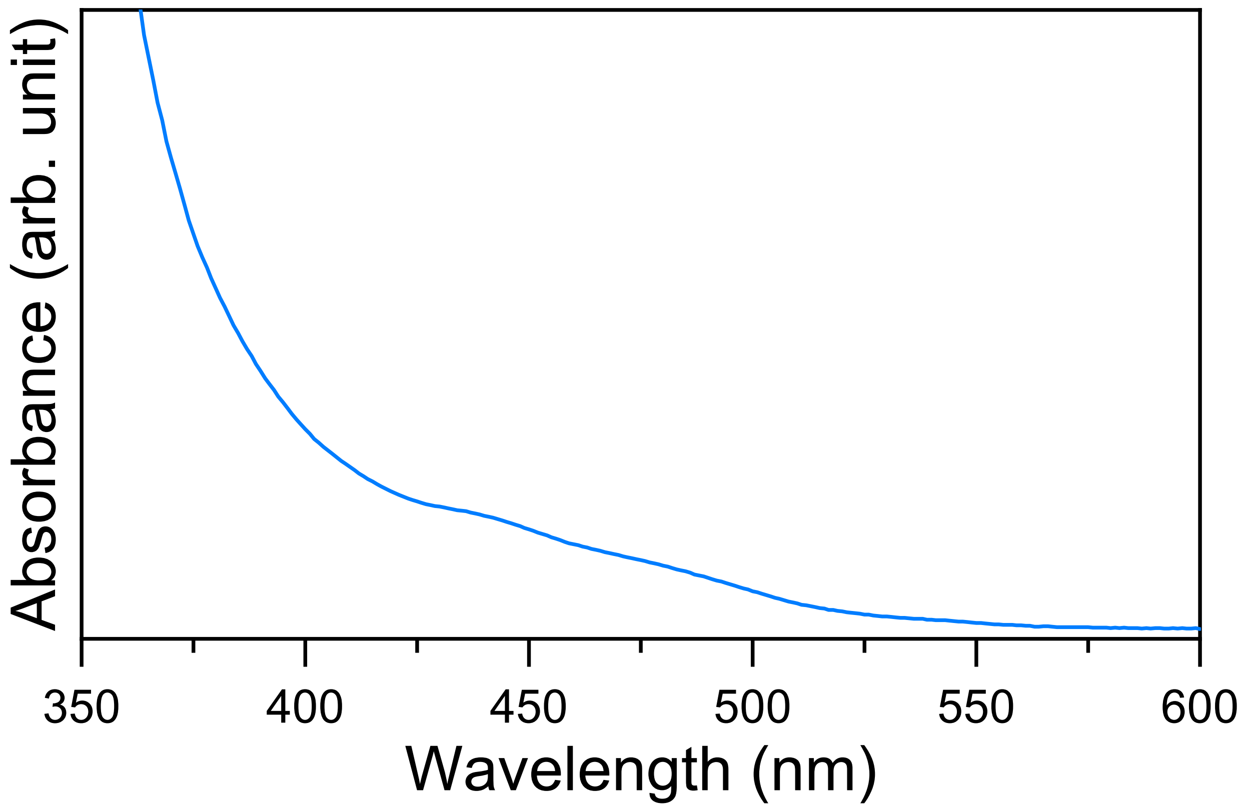

Figure S18: UV−vis spectrum of $[Co(bpy)_3]SO_4$ (1 mM) in KCl (50 mM) and $NaHCO_3$ (0.1 M) solution.

S13 Table of Z-Scheme H_2 Evolution Half Reactions

Table S1: Table of Z-scheme H_2 evolution half reactions.

Conditions	H_2 4 h (nmol)	TON 4 h	TOF 4 h (h^{-1})	Activity 4h (µmol h^{-1} g^{-1})
$SrTiO_3$:La,Rh|H_2ase+Cobpy	235.8 ± 24.6	11790 ± 1230	2947.5 ± 307.5	59.0 ± 6.2
$SrTiO_3$:La,Rh+Cobpy	44.5 ± 6.1	N.A.	N.A.	11.1 ± 1.5
$SrTiO_3$:La,Rh|H_2ase	2.7 ± 0.2	135 ± 10	33.8 ± 2.5	0.7 ± 0.05
H_2ase+Cobpy	1.8 ± 0.2	90 ± 10	22.5 ± 2.5	0.5 ± 0.05

S14 Table of Z-Scheme CO_2 Reduction Half Reactions

Table S2: Table of Z-scheme CO_2 reduction half reactions.

Conditions	Formate 4 h (nmol)	TON 4 h	TOF 4 h (h^{-1})	Activity 4 h (µmol h^{-1} g^{-1})
$SrTiO_3$:La,Rh|FDH+Cobpy	230.7 ± 31.0	4614 ± 620	1153.5 ± 155	57.7 ± 7.8
$SrTiO_3$:La,Rh+Cobpy	0	N.A.	N.A.	0
$SrTiO_3$:La,Rh|FDH	0	0	0	0
FDH+Cobpy	0	0	0	0

S15 Table of Z-Scheme Reactions

Table S3: Table of Z-scheme reactions.

Conditions	Product 10 h (nmol)	TON 10 h	TOF 10 h (h^{-1})	Activity 10 h (µmol h^{-1} g^{-1})
H_2ase|$SrTiO_3$:La,Rh|Cobpy|$BiVO_4$:Mo|RuO_2	1011.0 ± 71.3 H_2	50550 ± 3565	5055 ± 356.5	50.6 ± 3.6
FDH|$SrTiO_3$:La,Rh|Cobpy|$BiVO_4$:Mo|RuO_2	637 ± 54.8 formte	12740 ± 1096	1274 ± 109.6	31.9 ± 2.7

S16 Comparison among State-of-the-art $SrTiO_3$–$BiVO_4$ Z-Scheme Photocatalysts for Solar H_2 Evolution and CO_2 Reduction

Table S4: Comparison among state-of-the-art $SrTiO_3$–$BiVO_4$ Z-scheme photocatalysts for solar H_2 Evolution and CO_2 reduction.[S14–S21]

Z-Scheme System	Product	TOF (h^{-1})	Activity (μmol h^{-1} g^{-1})	Ref.
H_2ase|$SrTiO_3$:La,Rh|$[Co(bpy)_3]^{3+/2+}$|$BiVO_4$:Mo|RuO_2	H_2	5055	50.6	**This work**
FDH|$SrTiO_3$:La,Rh|$[Co(bpy)_3]^{3+/2+}$|$BiVO_4$:Mo|RuO_2	$HCOO^-$	1274	31.9	**This work**
Cr_2O_3/Ru|$SrTiO_3$:La,Rh|ITO|$BiVO_4$:Mo|RuO_2	H_2	90	5400	*Nat. Catal.* **2022**(S14)
S.ovata|Cr_2O_3/Ru|$SrTiO_3$:La,Rh|ITO|$BiVO_4$:Mo|RuO_2	CH_3COO^-	N.A.	1350	*Nat. Catal.* **2022**(S14)
CotpyP|$SrTiO_3$:La,Rh|Au|$BiVO_4$:Mo|RuO_2	$HCOO^-$	73.5	62.5	*Nat. Energy* **2020**(S15)
Au|$SrTiO_3$:Rh|$BiVO_4$	CO	0.02	0.2	*ACS AEM* **2020**(S16)
Cr_2O_3/Ru|$SrTiO_3$:La,Rh|ITO|$BiVO_4$:Mo|RuO_2	H_2	231	1950	*Joule* **2018**(S17)
Cr_2O_3/Ru|$SrTiO_3$:La,Rh|C|$BiVO_4$:Mo|RuO_X	H_2	325	3250	*J. Am. Chem. Soc.* **2017**(S18)
Cr_2O_3/Ru|$SrTiO_3$:La,Rh|Au|$BiVO_4$:Mo|RuO_X	H_2	450	4500	*Nat. Mater.* **2016**(S19)
Ru|$SrTiO_3$:Rh|$[Co(bpy)_3]^{3+/2+}$|$BiVO_4$	H_2	1.4	50	*J. Am. Chem. Soc.* **2013**(S20)
Ru|$SrTiO_3$:Rh|$Fe^{3+/2+}$|$BiVO_4$	H_2	0.2	2500	*J. Mater. Chem. A* **2013**(S21)

S17 Z-Scheme Photocatalysis under Visible Light

Z-scheme photocatalysis under visible light shows reduced performance compared to AM 1.5G illumination. This reduction is attributed to the absence of high-energy photons in the UV region of the Xe arc lamp irradiation spectrum, which corresponds to the UV region of the solar spectrum.

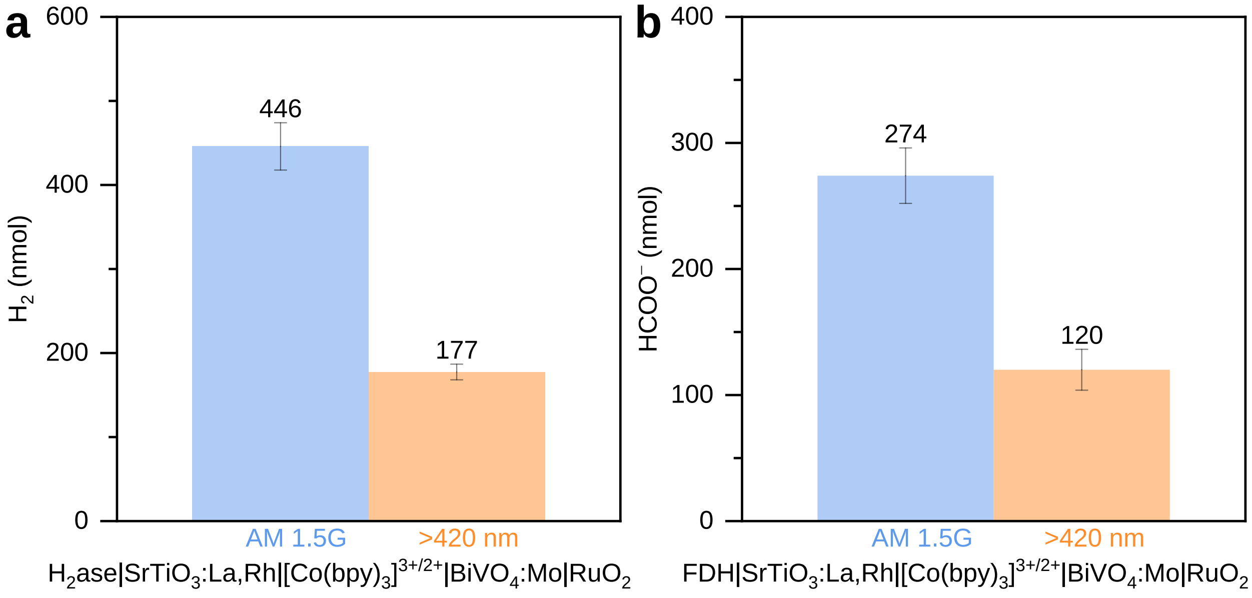

Figure S19: Z-scheme photocatalysis under visible light and AM 1.5G for (a) $H_2ase|SrTiO_3:La,Rh|[Co(bpy)_3]^{3+/2+}|BiVO_4:Mo|RuO_2$ and (b) $FDH|SrTiO_3:La,Rh|[Co(bpy)_3]^{3+/2+}|BiVO_4:Mo|RuO_2$. Conditions: a CO_2-saturated aqueous solution (1 mL, pH 6.7) containing $NaHCO_3$ (0.1 M), $[Co(bpy)_3]SO_4$ (0.5 mM), $SrTiO_3$:La,Rh (1 mg), $BiVO_4:Mo|RuO_2$ (1 mg), either H_2ase (20 pmol) or FDH (50 pmol), either AM 1.5G or >420 nm irradiation for 4 hours, 600 rpm stirring, 25 °C.

S18 Large Photoreactor and Closure Information

Each large photoreactor (Thermo Scientific, 10-SV) was sealed with a white rubber septum using the following procedure (Figure S20): 1) align a rubber septum with a clear glass photoreactor, 2) insert the serrated stopper of the septum into the photoreactor, 3) turnover the flange of the septum to grip outside of photoreactor neck to promote a double seal in conjunction.

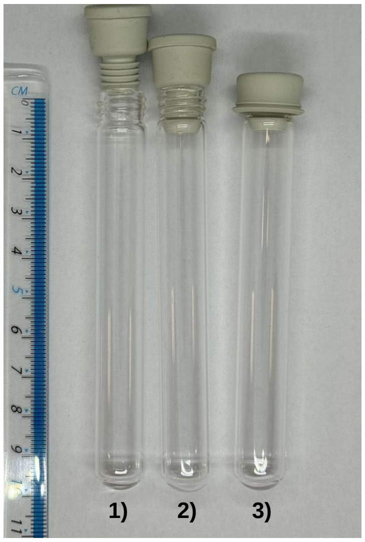

Photoreactor information	
Diameter	13 mm
Length	98 mm
Volume	10 mL
Base	round bottom
Closure	13-425 screw thread
Material	33 expansion borosilicate clear glass (Type 1, Class A)
Brand	Thermo Scientific™
Catalog no.	10-SV
Link	https://www.thermofisher.com/order/catalog/product/10-SV

Closure information	
Diameter (stopper)	9.5 mm
Diameter (flange)	16 mm
Length	21 mm
Fit	neck I.D. 9.5 mm for 10/30 joints
Material	white rubber
Brand	Sigma-Aldrich
Tradename	Suba-Seal® 13
Catalog no.	Z167258
Link	https://www.sigmaaldrich.com/GB/en/product/aldrich/z167258

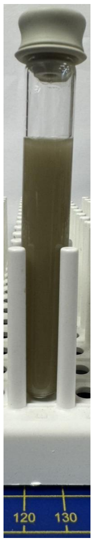

Figure S20: Left panel: photograph of the closure process of large photoreactors. Ruler unit: cm. Middle panel: specifications of the photoreactor and rubber septa. Right panel: photograph of an assembled Z-scheme photoreactor with 6 mL colloidal suspension. Grid unit: mm.

S19 Z-Scheme Photocatalysis with Large Photoreactors

Upon scaling the Z-scheme colloidal suspension by a factor of six, the production of H_2 and formate increased by factors of 4.2 and 4.9, respectively, when using H_2ase and FDH as co-catalysts. The mismatch in the scale-up factor between volumes and products is attributed to the challenges in maintaining turbulent flow in a larger stirred photoreactor, leading to partially stationary solutions. For fast turnover co-catalysts, such as molecular catalysts and enzymes, stationary solutions can cause mass transport limitations and local pH changes, resulting in reduced product yields.

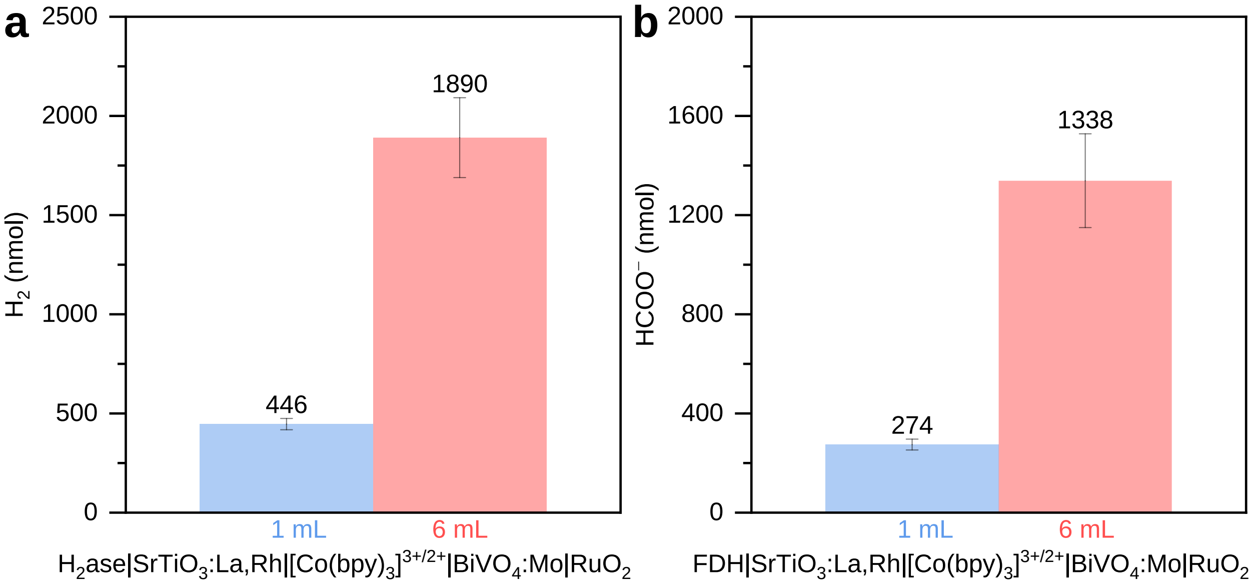

Figure S21: Z-scheme photocatalysis with large photoreactors for (a) H_2ase|$SrTiO_3$:La,Rh|$[Co(bpy)_3]^{3+/2+}$|$BiVO_4$:Mo|RuO_2 and (b) FDH|$SrTiO_3$:La,Rh|$[Co(bpy)_3]^{3+/2+}$|$BiVO_4$:Mo|RuO_2. Conditions: a CO_2-saturated aqueous solution (6 mL, pH 6.7) containing $NaHCO_3$ (0.1 M), $[Co(bpy)_3]SO_4$ (0.5 mM), $SrTiO_3$:La,Rh (6 mg), $BiVO_4$:Mo|RuO_2 (6 mg), either H_2ase (120 pmol) or FDH (300 pmol), AM 1.5G irradiation for 4 hours, 600 rpm stirring, 25 °C.

S20 Isotopic Labeling Experiments

Photocatalysis experiments were carried out in either $NaH^{12}CO_3$ (0.1 M) aqueous solution with $^{12}CO_2$ as the headspace gas or $NaH^{13}CO_3$ (0.1 M) aqueous solution with $^{13}CO_2$ as the headspace gas. After 10 h of simulated AM 1.5G irradiation, the solution was transferred to an NMR tube and ^{1}H NMR spectra were collected with a 400 MHz NMR spectrometer. ^{1}H NMR spectra of commercial sodium formate-^{12}C ($H^{12}COONa$) and sodium formate-^{13}C ($H^{13}COONa$) were recorded to compare with the ^{1}H NMR spectra of the labeled products. All ^{1}H NMR spectra are shown in Figure S22.

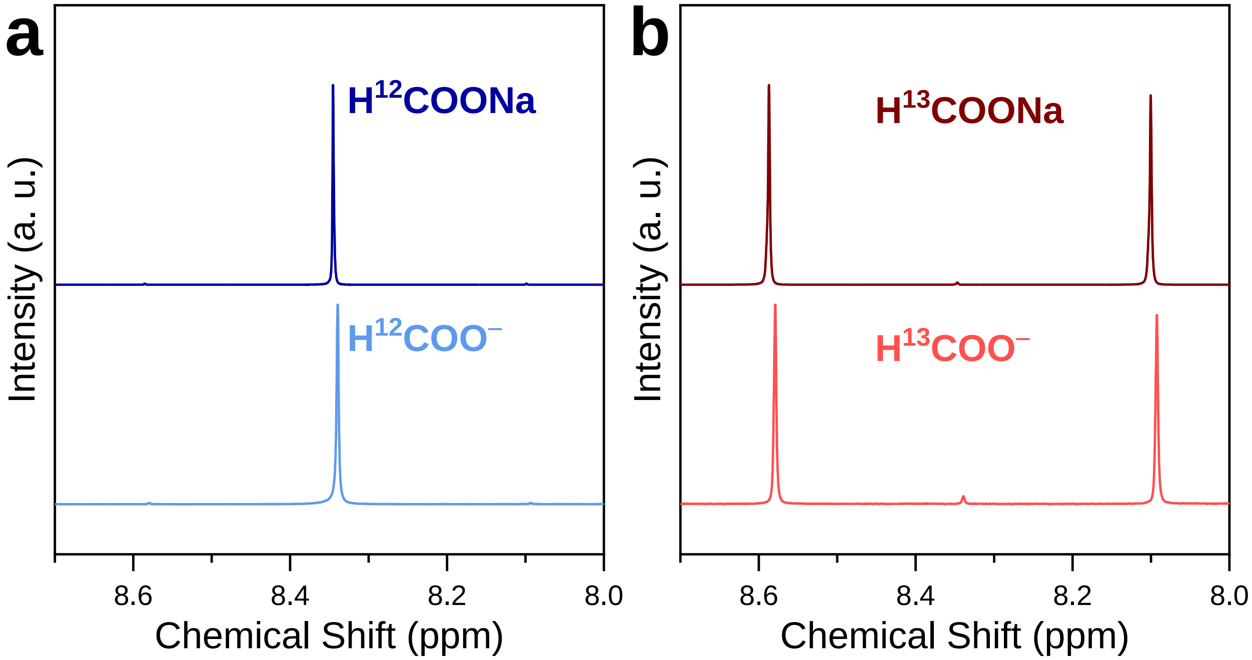

Figure S22: ^{1}H NMR spectra of (a) 10 h photocatalysis of $NaH^{12}CO_3$ aqueous solution with $^{12}CO_2$ headspace and (b) 10 h photocatalysis of $NaH^{13}CO_3$ aqueous solution with $^{13}CO_2$ headspace. Conditions: a CO_2-saturated aqueous solution (1 mL, pH 6.7) containing $NaHCO_3$ (0.1 M), $[Co(bpy)_3]SO_4$ (0.5 mM), $SrTiO_3$:La,Rh (1 mg), $BiVO_4$:Mo|RuO_2 (1 mg), FDH (50 pmol), AM 1.5G irradiation, 600 rpm stirring, 25 °C. ^{1}H NMR spectra of commercial (a) sodium formate-^{12}C ($H^{12}COONa$) and (b) sodium formate-^{13}C ($H^{13}COONa$) were recorded as a comparison.

S21 Table of PEIS Fitting Results

Table S5: Table of PEIS fitting results. Error bars represent the fitting errors.

Working electrode	R_S (Ω)	R_{ct} (Ω)	C_{bulk} (μF)
$SrTiO_3$:La,Rh	190.1 ± 1.5	15724 ± 155	24.6 ± 0.3
$SrTiO_3$:La,Rh|FDH	188.6 ± 1.8	8713 ± 90	23.8 ± 0.4
$SrTiO_3$:La,Rh|H_2ase	187.2 ± 2.8	6747 ± 93	19.4 ± 0.6
$BiVO_4$:Mo	215.3 ± 4.9	153280 ± 19439	6.3 ± 0.2
$BiVO_4$:Mo|RuO_2	226.6 ± 3.0	79076 ± 6905.1	13.6 ± 0.3

S22 Table of IMVS Fitting Results

Table S6: Table of IMVS fitting results. Error bars represent the fitting errors.

Working electrode	V_{SS} (mV)	τ_n (μs)	α
$SrTiO_3$:La,Rh	1.23 ± 0.005	466 ± 7.0	0.80 ± 0.01
$SrTiO_3$:La,Rh|FDH	1.73 ± 0.003	412 ± 10.0	0.83 ± 0.01
$SrTiO_3$:La,Rh|H_2ase	2.00 ± 0.007	379 ± 12.0	0.85 ± 0.02

S23 Impedance Analysis on the Oxidation Half Reaction

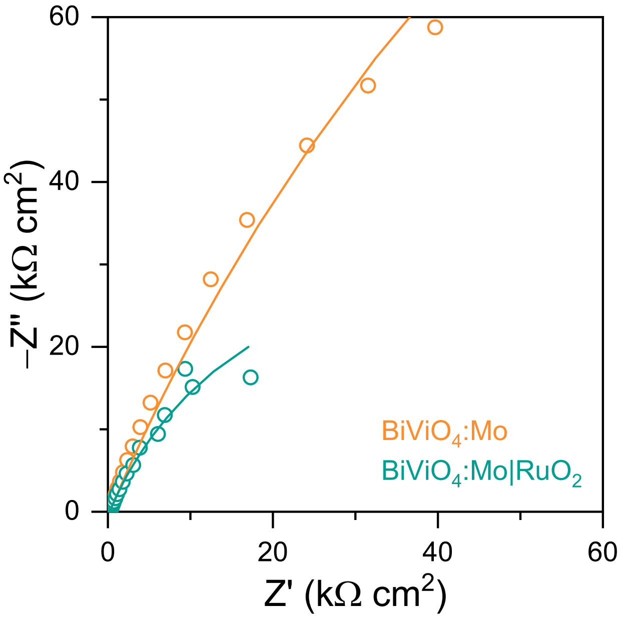

Figure S23: Nyquist plots of PEIS response recorded at −0.2 V vs. RHE (open circuits) with corresponding fitted curves (solid lines). Conditions: a CO_2-saturated aqueous solution (20 mL, pH 6.7) containing $NaHCO_3$ (0.1 M), $[Co(bpy)_3]^{3+/2+}$ (0.5 mM) and KCl (50 mM), $BiVO_4$:Mo and $BiVO_4$:Mo|RuO_2 working electrodes, Ag/AgCl (sat. KCl) reference electrode, Pt mesh counter electrode, AM 1.5G irradiation, 25 °C.

S24 Small Photoreactor and Closure Information

Each small photoreactor (Thermo Scientific, 5-SV) was sealed with a white rubber septum using the following procedure (Figure S24): 1) align the rubber septum with a clear glass photoreactor, 2) insert the serrated stopper of the septum into the photoreactor, 3) turnover the flange of the septum to grip outside of photoreactor neck to promote a double seal in conjunction.

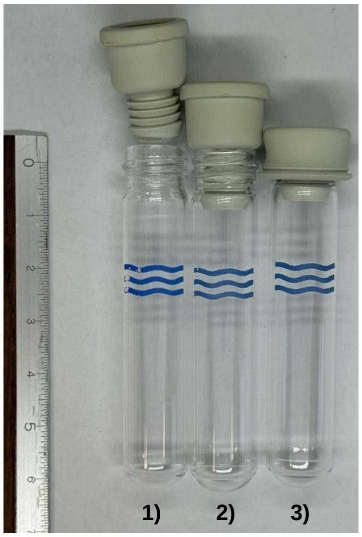

Photoreactor information	
Diameter	13 mm
Length	65 mm
Volume	5 mL
Base	round bottom
Closure	13-425 screw thread
Material	33 expansion borosilicate clear glass (Type 1, Class A)
Brand	Thermo Scientific™
Catalog no.	5-SV
Link	https://www.thermofisher.com/order/catalog/product/5-SV

Closure information	
Diameter (stopper)	9.5 mm
Diameter (flange)	16 mm
Length	21 mm
Fit	neck I.D. 9.5 mm for 10/30 joints
Material	white rubber
Brand	Sigma-Aldrich
Tradename	Suba-Seal® 13
Catalog no.	Z167258
Link	https://www.sigmaaldrich.com/GB/en/product/aldrich/z167258

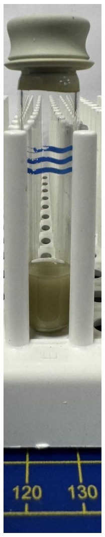

Figure S24: Left panel: photograph of the closure process of small photoreactors. Ruler unit: cm. Middle panel: specifications of the photoreactor and rubber septa. Right panel: photograph of an assembled Z-scheme photoreactor with 1 mL colloidal suspension. Grid unit: mm.

S25 Light Source Setup

Photoreactors were positioned in front of the irradiation source (Figure S25a), where the irradiance at the photoreactor position was AM 1.5G (100 mW cm^{-2}). To measure the irradiance at the photoreactor position, a Newport model 919P-020-12 thermopile sensor with an active area of 1.13 cm^2 (Figure S25b) was used, connected to a Newport model 853-R optical power meter (Figure S25c). In the current experimental setup, the optical power meter displayed a reading of 0.113 W (Figure S25c), which corresponds to an irradiance of 100 mW cm^{-2} (AM 1.5G). The distance between the photoreactor and the lens was 17.6 cm, and the distance between the lens and the light source was 15.5 cm (Figure S25a).

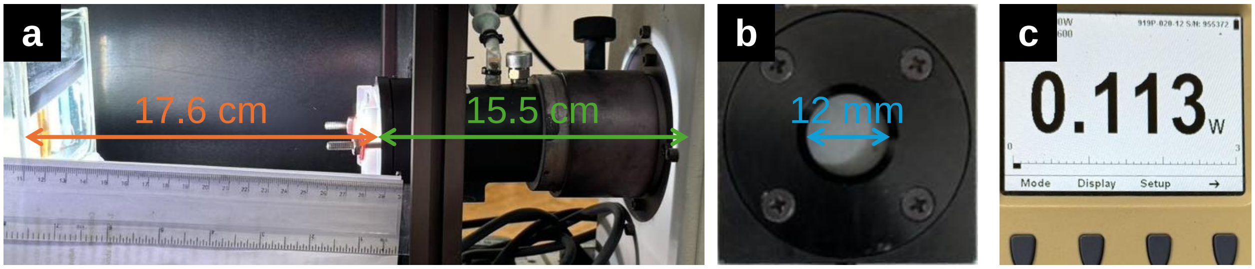

Figure S25: Photograph of (a) lamp distance setup for photocatalysis, (b) thermopile sensor for the optical power meter, and (c) optical power meter reading at the photoreactor position.

S26 Photograph of Electrodes

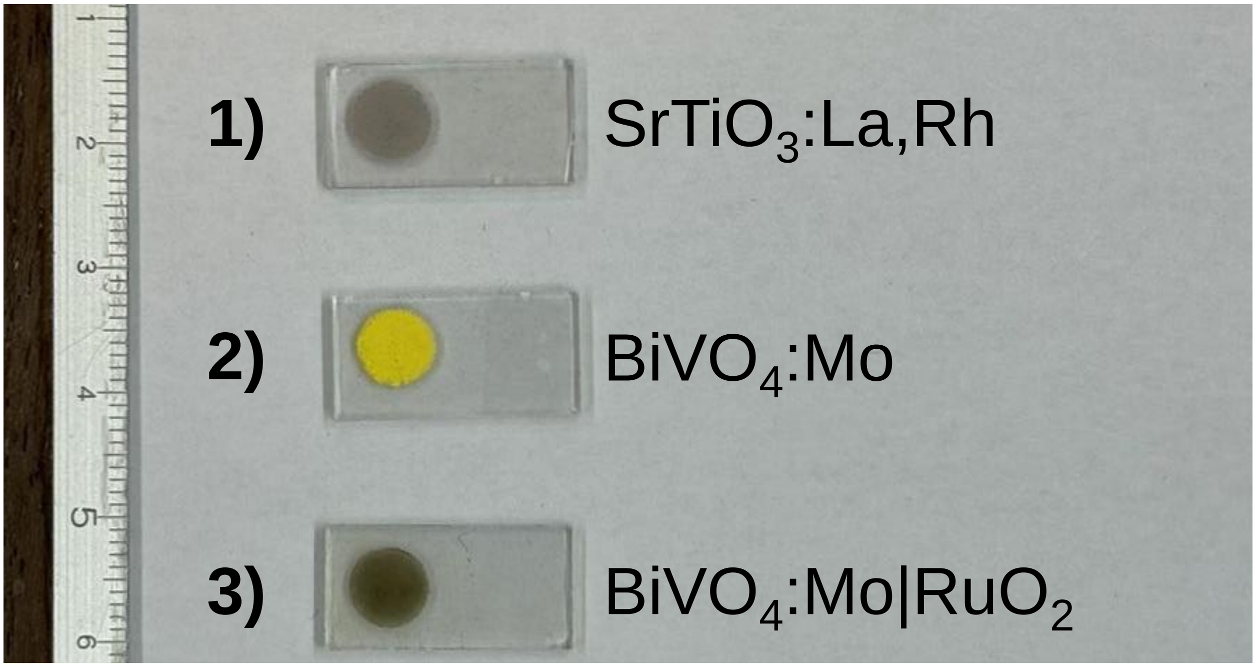

Figure S26: Photograph of 1) $SrTiO_3$:La,Rh, 2) $BiVO_4$:Mo, and 3) $BiVO_4$:Mo|RuO_2 electrodes with 0.25 cm^2 active area. Ruler unit: cm.

References

(S1) Makuła, P.; Pacia, M.; Macyk, W. How to correctly determine the band gap energy of modified semiconductor photocatalysts based on UV–Vis spectra. *J. Phys. Chem. Lett.* **2018**, *9*, 6814–6817.

(S2) Sivula, K. Mott–Schottky analysis of photoelectrodes: sanity checks are needed. *ACS Energy Lett.* **2021**, *6*, 2549–2551.

(S3) Rettie, A. J.; Lee, H. C.; Marshall, L. G.; Lin, J.-F.; Capan, C.; Lindemuth, J.; McCloy, J. S.; Zhou, J.; Bard, A. J.; Mullins, C. B. Combined charge carrier transport and photoelectrochemical characterization of $BiVO_4$ single crystals: intrinsic behavior of a complex metal oxide. *J. Am. Chem. Soc.* **2013**, *135*, 11389–11396.

(S4) Yang, Z.; Lee, D.; Yue, J.; Gabel, J.; Lee, T.-L.; James, R. D.; Chambers, S. A.; Jalan, B. Epitaxial $SrTiO_3$ films with dielectric constants exceeding 25,000. *Proc. Natl. Acad. Sci. U. S. A.* **2022**, *119*, e2202189119.

(S5) Wunderlich, W.; Ohta, H.; Koumoto, K. Enhanced effective mass in doped $SrTiO_3$ and related perovskites. *Physica B* **2009**, *404*, 2202–2212.

(S6) Walsh, A.; Yan, Y.; Huda, M. N.; Al-Jassim, M. M.; Wei, S.-H. Band edge electronic structure of $BiVO_4$: elucidating the role of the Bi s and V d orbitals. *Chem. Mater.* **2009**, *21*, 547–551.

(S7) Sze, S. M.; Li, Y.; Ng, K. K. *Physics of Semiconductor Devices*; John Wiley & Sons, 2021.

(S8) Wang, Q.; Hisatomi, T.; Ma, S. S. K.; Li, Y.; Domen, K. Core/shell structured La-and Rh-codoped $SrTiO_3$ as a hydrogen evolution photocatalyst in Z-scheme overall water splitting under visible light irradiation. *Chem. Mater.* **2014**, *26*, 4144–4150.

(S9) Wu, Q.; Xiao, M.; Wang, W.; Cui, C. In situ coordination environment tuning of cobalt sites for efficient water oxidation. *ACS Catal.* **2019**, *9*, 11734–11742.

(S10) Lin, J.; Hou, Y.; Zheng, Y.; Wang, X. Integration of $[(Co(bpy)_3]^{2+}$ electron mediator with heterogeneous photocatalysts for CO_2 conversion. *Chem. Asian J.* **2014**, *9*, 2468–2474.

(S11) Yamaguchi, K.; Kume, S.; Namiki, K.; Murata, M.; Tamai, N.; Nishihara, H. UV−Vis, NMR, and Time-Resolved Spectroscopy Analysis of Photoisomerization Behavior of Three-and Six-Azobenzene-Bound Tris (bipyridine) cobalt Complexes. *Inorg. Chem.* **2005**, *44*, 9056–9067.

(S12) Strukl, J.; Walter, J. Infrared and Raman spectra of heterocyclic compounds—III: The infrared studies and normal vibrations of 2,2'-bipyridine. *Spectrochim. Acta A* **1971**, *27*, 209–221.

(S13) Gerasimova, T. P.; Katsyuba, S. A. Bipyridine and phenanthroline IR-spectral bands as indicators of metal spin state in hexacoordinated complexes of Fe (II), Ni (II) and Co (II). *Dalton Trans.* **2013**, *42*, 1787–1797.

(S14) Wang, Q.; Kalathil, S.; Pornrungroj, C.; Sahm, C. D.; Reisner, E. Bacteria–photocatalyst sheet for sustainable carbon dioxide utilization. *Nat. Catal.* **2022**, *5*, 633–641.

(S15) Wang, Q.; Warnan, J.; Rodríguez-Jiménez, S.; Leung, J. J.; Kalathil, S.; Andrei, V.; Domen, K.; Reisner, E. Molecularly engineered photocatalyst sheet for scalable solar formate production from carbon dioxide and water. *Nat. Energy* **2020**, *5*, 703–710.

(S16) Yoshino, S.; Sato, K.; Yamaguchi, Y.; Iwase, A.; Kudo, A. Z-schematic CO_2 reduction to CO through interparticle electron transfer between $SrTiO_3$:Rh of a reducing photocatalyst and $BiVO_4$ of a water oxidation photocatalyst under visible light. *ACS Appl. Energy Mater.* **2020**, *3*, 10001–10007.

(S17) Wang, Q.; Okunaka, S.; Tokudome, H.; Hisatomi, T.; Nakabayashi, M.; Shibata, N.; Yamada, T.; Domen, K. Printable photocatalyst sheets incorporating a transparent conductive mediator for Z-scheme water splitting. *Joule* **2018**, *2*, 2667–2680.

(S18) Wang, Q.; Hisatomi, T.; Suzuki, Y.; Pan, Z.; Seo, J.; Katayama, M.; Minegishi, T.; Nishiyama, H.; Takata, T.; Seki, K.; Kudo, A.; Yamada, T.; Domen, K. Particulate photocatalyst sheets based on carbon conductor layer for efficient Z-scheme pure-water splitting at ambient pressure. *J. Am. Chem. Soc.* **2017**, *139*, 1675–1683.

(S19) Wang, Q.; Hisatomi, T.; Jia, Q.; Tokudome, H.; Zhong, M.; Wang, C.; Pan, Z.; Takata, T.; Nakabayashi, M.; Shibata, N.; Li, Y.; Sharp, I. D.; Kudo, A.; Yamada, T.; Domen, K. Scalable water splitting on particulate photocatalyst sheets with a solar-to-hydrogen energy conversion efficiency exceeding 1%. *Nat. Mater.* **2016**, *15*, 611–615.

(S20) Sasaki, Y.; Kato, H.; Kudo, A. $[Co(bpy)_3]^{3+/2+}$ and $[Co(phen)_3]^{3+/2+}$ electron mediators for overall water splitting under sunlight irradiation using Z-scheme photocatalyst system. *J. Am. Chem. Soc.* **2013**, *135*, 5441–5449.

(S21) Kato, H.; Sasaki, Y.; Shirakura, N.; Kudo, A. Synthesis of highly active rhodium-doped $SrTiO_3$ powders in Z-scheme systems for visible-light-driven photocatalytic overall water splitting. *J. Mater. Chem. A* **2013**, *1*, 12327–12333.
